# Defects in the mitochondrial-tRNA modification enzymes MTO1 and GTPBP3 promote different metabolic reprogramming through a HIF-PPARγ-UCP2-AMPK axis

**DOI:** 10.1038/s41598-018-19587-5

**Published:** 2018-01-18

**Authors:** Rachid Boutoual, Salvador Meseguer, Magda Villarroya, Elena Martín-Hernández, Mohammed Errami, Miguel A. Martín, Marta Casado, M.-Eugenia Armengod

**Affiliations:** 1 RNA Modification and Mitochondrial Diseases Laboratory, Centro de Investigación Príncipe Felipe (CIPF), Valencia, 46012 Spain; 2 Faculty of Sciences, Abdelmalek Essaadi University, Tetouan, BP.2121 Morocco; 3 Unidad de Enfermedades Mitocondriales y Enfermedades Metabólicas Hereditarias, Departamento de Pediatría, Hospital 12 de Octubre, Madrid, 28041 Spain; 4 Mitochondrial and Neuromuscular Disorders Laboratory, Hospital Universitario 12 de Octubre, Madrid, 28041 Spain; 5 Centro de Investigación Biomédica en Red de Enfermedades Raras (CIBERER) nodo U723, Madrid, 28029 Spain; 6 Instituto de Biomedicina de Valencia, IBV-CSIC, Valencia, 46010 Spain; 7 Centro de Investigación Biomédica en Red de Enfermedades Hepáticas y Digestivas (CIBERehd), and Centro de Investigación Biomédica en Red de Enfermedades Cardiovasculares (CIBERcv), Madrid, 28029 Spain; 8 Centro de Investigación Biomédica en Red de Enfermedades Raras (CIBERER) node 721, Madrid, 28029 Spain

## Abstract

Human proteins MTO1 and GTPBP3 are thought to jointly catalyze the modification of the wobble uridine in mitochondrial tRNAs. Defects in each protein cause infantile hypertrophic cardiomyopathy with lactic acidosis. However, the underlying mechanisms are mostly unknown. Using fibroblasts from an MTO1 patient and MTO1 silenced cells, we found that the MTO1 deficiency is associated with a metabolic reprogramming mediated by inactivation of AMPK, down regulation of the uncoupling protein 2 (UCP2) and transcription factor PPARγ, and activation of the hypoxia inducible factor 1 (HIF-1). As a result, glycolysis and oxidative phosphorylation are uncoupled, while fatty acid metabolism is altered, leading to accumulation of lipid droplets in MTO1 fibroblasts. Unexpectedly, this response is different from that triggered by the GTPBP3 defect, as GTPBP3-depleted cells exhibit AMPK activation, increased levels of UCP2 and PPARγ, and inactivation of HIF-1. In addition, fatty acid oxidation and respiration are stimulated in these cells. Therefore, the HIF-PPARγ-UCP2-AMPK axis is operating differently in MTO1- and GTPBP3-defective cells, which strongly suggests that one of these proteins has an additional role, besides mitochondrial-tRNA modification. This work provides new and useful information on the molecular basis of the MTO1 and GTPBP3 defects and on putative targets for therapeutic intervention.

## Introduction

Mitochondria play crucial roles in energy production, metabolic pathways, cell signaling and apoptosis. They produce most of the cellular ATP via oxidative phosphorylation (OXPHOS), which occurs in the inner mitochondrial membrane and requires more than 100 proteins organized into five multimeric complexes and two mobile electron shuttles, coenzyme Q (CoQ) and cytochrome *c*. Complexes I to IV form the mitochondrial respiratory chain, which transfers electrons from reducing equivalents (NADH and FADH_2_) to molecular oxygen, creating a proton gradient across the inner mitochondrial membrane that is used by complex V to synthesize ATP. NADH and FADH_2_ are produced by different metabolic pathways, including tricarboxylic acid (TCA) cycle and oxidation of fatty acids. While NADH reducing equivalents are funnelled into the mitochondrial electron transport chain through complex I, FADH_2_ reducing equivalents are incorporated through complex II or diverse electron transfer flavoproteins.

Thirteen key OXPHOS subunits are encoded by the mitochondrial DNA (mtDNA), which also encodes the 22 tRNAs and 2 rRNAs responsible for the intra-mitochondrial protein synthesis. The rest of factors required for normal mitochondrial protein synthesis, such as mitoribosomal proteins, translation factors, aminoacyl tRNA synthetases and RNA modification enzymes, are encoded by the nuclear genome (nDNA). Defects in mitochondrial translation due to mutations in either mtDNA or nDNA cause diseases that are usually associated with OXPHOS dysfunction and a variety of tissue-specific clinical presentations^1,2^. In particular, mutations in the nuclear genes *GTPBP3* (MIM #608536) and *MTO1* (MIM #614667), which encode proteins involved in the post-transcriptional modification of a mitochondrial-tRNA (mt-tRNA) group, cause infantile hypertrophic cardiomyopathy with lactic acidosis and, quite often, neurological symptoms^3–9^. From studies of their bacterial and yeast orthologs, proteins GTPBP3 and MTO1 are predicted to jointly catalyze the addition of the taurinomethyl group at position 5 of the anticodon wobble uridine (U34) in mt-tRNAs decoding for Lys, Glu, Gln, Leu^(UUR)^, and Trp^10,11^. A third nuclear-encoded protein named TRMU or MTU1 (which is also conserved from bacteria to humans) thiolates position 2 of U34 in a subset of these mt-tRNAs (those decoding for Lys, Glu and Gln)^12–16^. Curiously, mutations in *TRMU* (MIM #610230), although also leading to OXPHOS dysfunction, cause liver failure^12,14^. The reasons why *TRMU* and *GTPBP3* or *MTO1* mutations produce different clinical outcomes is currently unknown. We have proposed that these defects affect retrograde signalling from mitochondria to nucleus in a different manner, which results in different tissue-dependent nuclear responses and, consequently, in different phenotypes^17^. In this respect, we have reported that stable silencing of *GTPBP3* triggers an AMPK-dependent retrograde signalling pathway, which down-regulates the mitochondrial pyruvate carrier (MPC), while up-regulating the expression of the uncoupling protein 2 (UCP2) and genes involved in glycolysis and fatty acid oxidation^18^. These data suggest that the GTPBP3 defect promotes, in the cell model, a shift from pyruvate to fatty acid oxidation, and leads to an uncoupling of glycolysis and oxidative phosphorylation.

In this work, we explore the cell response to MTO1 deficiency by using fibroblasts from a patient carrying the *MTO1* mutation c.1392 C>T (p.Arg464Cys) in homozygosis^9^ and MTO1-silenced cells. Moreover, we compare the molecular findings in both types of cells with those obtained from GTPBP3-silenced cells. We present evidences that mutation p.Arg464Cys or MTO1-depletion severely affects both the tRNA modification activity of MTO1 and key aspects of the mitochondrial function. Our data indicate that the MTO1 deficiency down regulates the expression of a PPARγ/UCP2/AMPK axis, which results in fatty acid oxidation impairment and intracellular lipid accumulation. Strikingly, these effects are different from those found after stable or transient silencing of *GTPBP3*, represented by an increased signalling from the PPARγ-UCP2-AMPK axis. Altogether our data suggest that MTO1 or GTPBP3 has an additional role besides mt-tRNA modification. Notably, the phenotype associated with the MTO1 defect can be partially reversed by treatment with Rosiglitazone, a PPARγ agonist, or AICAR, an AMPK activator, suggesting that the components of the PPARγ-UCP2-AMPK axis are putative targets for therapeutic intervention.

## Results

### **Mutation c.1392 C**>T (p.Arg464Cys) affects the mt-tRNA modification activity of MTO1 but not its cellular localization

Arg464 is a strictly conserved residue equivalent to Arg427 in the *E. coli* MnmG protein (the MTO1 *E.coli* homologue), where the change to Ala has been shown to decrease the tRNA modification activity^19,20^. In order to explore the effect of the clinical pArg464Cys mutation on the tRNA modification function of the protein, we followed two approaches. On one hand, we analysed the capability of the *E. coli* MnmG protein carrying the Arg427Cys change to modify bacterial tRNAs *in vivo*. HPLC analysis of the nucleoside composition of total tRNA purified from a strain expressing MnmG-Arg427Cys indicated that this protein was unable to modify *E. coli* tRNAs (Table S1 and Fig. S1A). Of note, western blot analysis revealed that the protein stability was not affected by the Arg427Cys change (Fig. S1B). Considering the strict evolutionary conservation of this arginine residue, it is reasonable to conclude that the human mutation p.Arg464Cys will affect the modification activity of MTO1.

**Figure 1:**
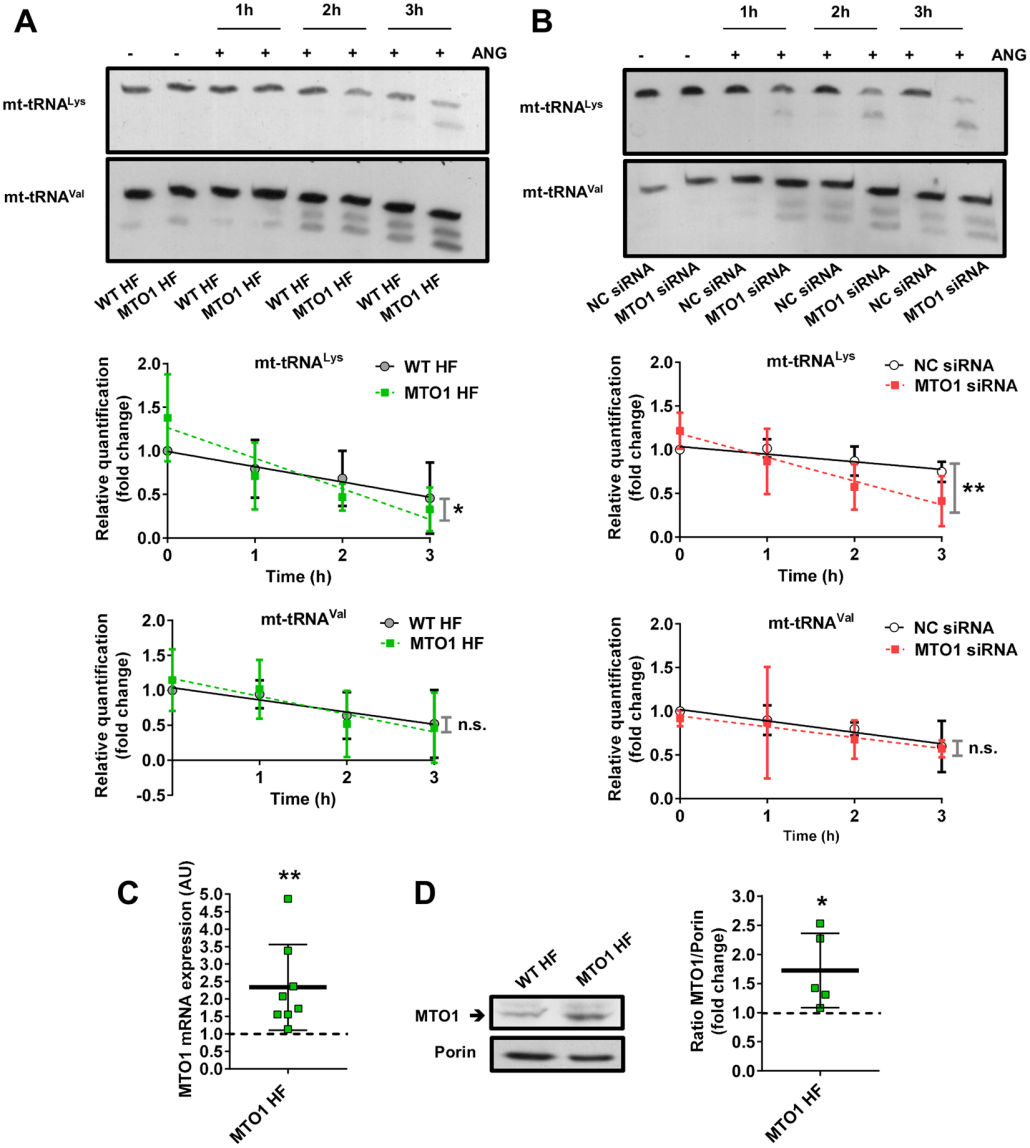
Angiogenin sensitivity of mt-tRNAs purified from MTO1 defective cells. **(A** and **B)** Northern analysis of mt-tRNA^Lys^ (upper panel) and mt-tRNA^Val^ (lower panel) molecules after *in vitro* angiogenin (ANG) digestion of small RNAs purified from wild-type (WT HF) and MTO1 (MTO1 HF) human fibroblasts (**A**), and from MTO1 siRNA 1- and Negative Control (NC) siRNA-transfected 143B cells (**B**) for 1, 2 and 3 h. Full-length blots are included in supplementary information (Fig. S17). The kinetic analysis of angiogenin digestions are plotted below the representative northern blots. The amount of intact mt-tRNA after 0, 1, 2 and 3 h of incubation with angiogenin is represented as fold change relative to the undigested control (0 h). (**C)** qRT-PCR analysis of *MTO1* mRNA in MTO1 HF. Data are expressed as fold change respect to WT HF. **(D)** Representative immunoblot of MTO1 protein expression in MTO1 HF and WT HF. The membrane was also probed with an antibody against porin, which was used as a loading control. Full-length western blots are included in supplementary information (Fig. S18). The scatter plot shows the densitometric analysis of MTO1 normalized to the loading control and represented as fold change respect to WT HF. All data are the mean ± SD of at least three independent biological replicates. Differences from WT or NC values were found to be statistically significant at *p < 0.05 and **p < 0.01. n.s.: non-significant differences.

On the other hand, given the difficulty in obtaining enough amounts of mt-tRNAs from fibroblasts for nucleoside analysis by HPLC or mass-spectrometry, we determined the sensitivity of mt-tRNAs from patient fibroblasts to digestion with the tRNA-specific RNase angiogenin (ANG)^21,22^. This approach was based on previous findings indicating that loss of the U34 modification at position 5 increases the angiogenin-mediated cleavage of the *E. coli* tRNA^Lys^, which is a substrate for the MTO1 and GTPBP3 bacterial orthologs^18^. This qualitative approach has proven to be useful in analysing the modification status of mt-tRNAs obtained from GTPBP3 knocked-down cells^18^ and *Caenorhabditis elegans* strains carrying a deletion mutation in the *GTPBP3* or *MTO1* homolog^17^. As shown in Fig. 1A, mt-tRNA^Lys^ purified from MTO1 fibroblasts was more sensitive to angiogenin-mediated digestion than mt-tRNA^Lys^ obtained from the control cells. In contrast, we found no differences in the digestion patterns of a non-substrate tRNA of MTO1 (mt-tRNA^Val^). These data support the idea that the p.Arg464Cys mutation affects the modification activity of MTO1. We also found that the angiogenin-sensitivity of mt-tRNA^Lys^ obtained from wild-type 143B cells was increased after down-regulation of *MTO1* by siRNAs (Fig. 1B), which were shown to reduce the MTO1 expression by about 50% (Fig. S2A and B). Altogether these data indicate that either low activity or levels of MTO1 result in hypomodification of its mt-tRNA substrates.

It should be mentioned that an overexpressed MTO1-Arg464Cys protein exhibited the typical mitochondrial localization pattern (Fig. S3). Moreover, qRT-PCR and western blot analysis indicated that the MTO1 mRNA and protein levels were higher in patient fibroblasts (MTO1 fibroblasts) than in control cells (Fig. 1C and D). These results indicate that the mt-tRNA^Lys^ hypomodification detected in MTO1 fibroblasts is due to the inactivation of the MTO1 function rather than to a mislocalization or a lower expression of the mutant MTO1 protein. The increased expression of the mutant protein in patient fibroblasts (Fig. 1C and D) suggests that regulation of *MTO1* involves adaptive mechanisms aimed to increase the MTO1 steady-state levels in an attempt to compensate for a deficit of the MTO1 function.

### MTO1 defective cells exhibit proteostasis stress and an altered bioenergetic state

**Figure 2:**
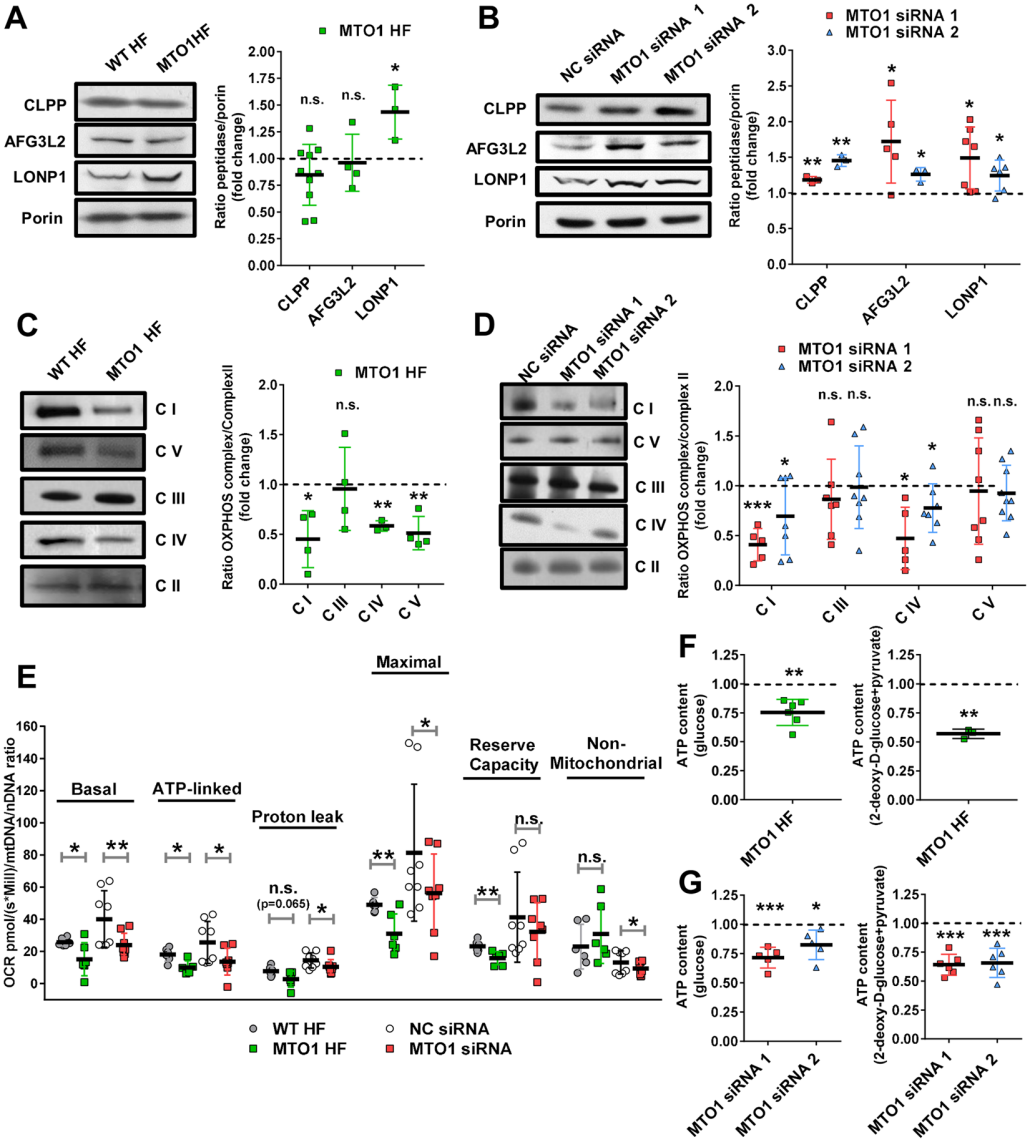
MTO1 defective cells exhibit proteostasis stress and an altered bioenergetic state. **(A** and **B)** Representative immunoblots showing the expression of CLPP, AFG3L2 and LONP1 in extracts of WT and MTO1 HF (**A**), and in MTO1 siRNA 1-, MTO1 siRNA 2- and Negative Control (NC) siRNA-transfected 143B cells (**B**). Porin was used as a loading control. Full-length blots are included in supplementary information (Fig. S19). The scatter plots show the densitometric measurements of the mitoproteases normalized to the loading control and represented as fold change relative to control cells. **(C** and **D)** Representative Blue Native-PAGE of OXPHOS complexes in WT and MTO1 HF (**C**), and in MTO1 siRNA 1-, MTO1 siRNA 2- and NC siRNA-transfected 143B cells (**D**). Full-length blots and lower-exposure blots of complex III are included in supplementary information (Fig. S20). The scatter plots show the densitometric measurements of OXPHOS complexes normalized to complex-II (loading control) and represented as fold change relative to control cells. **(E)** Analysis of oxygen consumption rate (OCR) of intact cells using different OXPHOS inhibitors. OCR was measured in each cell type under basal conditions and after sequential addition of oligomycin, carbonyl cyanide-p-trifluoromethoxyphenylhydrazone (CCCP), rotenone and antimycin A. The scatter plot shows basal OCR (determined as the difference between OCR before oligomycin and OCR after rotenone/antimycin A), ATP-linked OCR (difference between OCR before and after oligomycin), proton leak (difference between basal OCR and ATP-linked OCR), reserve capacity (difference between the CCCP-stimulated rate and basal OCR), non-mitochondrial OCR (OCR after rotenone and antimycin A treatment), and maximal OCR (difference between OCR after CCCP and non-mitochondrial OCR). **(F** and **G)** Measurement of total and mitochondrial ATP levels in fibroblasts (**F**) and MTO1-silenced 143B cells (**G**). Cells were incubated with 5 mM glucose or 2.5 mM 2-deoxy-d-glucose plus 2.5 mM pyruvate (left and right, respectively) to determine total and mitochondrial ATP levels. Data are expressed as fold change respect to WT HF (**F**) or NC siRNA-transfected 143B cells (**G**) values. All data are the mean ± SD of at least three different experiments. Differences from control values were found to be statistically significant at *p < 0.05, **p < 0.01, ***p < 0.001. n.s.: non-significant differences.

mt-tRNA hypomodification due to MTO1 defects has been proposed to impair mitochondrial translation and lead to disruption of the stoichiometric balance between components of OXPHOS complexes, unleashing proteostasis stress^8^. Common markers of this stress are mitoproteases like LONP1, CLPP or AFG3L2^23–31^. We detected an increase of the LONP1 levels in MTO1 fibroblasts (Fig. 2A), and increased levels of the three proteases in MTO1-silenced 143B cells (Fig. 2B). Therefore, these data support the previous proposal that a deficit of the MTO1 function produces proteostasis stress^8^. The different protease response between MTO1 fibroblasts and the MTO1-silenced cells may be a consequence of differences in the cell type, genetic/epigenetic background, and/or the acute MTO1 fail in the case of the silenced cells.

Blue native polyacrylamide gel electrophoresis (BN-PAGE) and subsequent western blot analysis of fibroblast extracts revealed a reduction in the steady-state levels of complexes I, IV and V (Fig. 2C). Notably, a decrease in the activities of complex I and IV was detected in a proband´s muscle biopsy, which also showed deficiency in complex III but increased complex II + III coupled activity (Table S2). Alterations of OXPHOS complexes in MTO1 fibroblasts were accompanied with a reduction of the steady-state levels of several nuclear- and mitochondrial-encoded OXPHOS subunits (Fig. S4). Reduced steady-state levels of complexes I and IV was also observed in MTO1-depleted 143B cells (Fig. 2D).

We also investigated the impairment of the OXPHOS function in MTO1-deficient cells by measuring the oxygen consumption rate (OCR). We found that basal, ATP-linked, maximal OCR, and reserve respiratory capacity were significantly decreased in MTO1 fibroblasts with respect to control cells (Fig. 2E). A decrease in the mitochondrial proton leak was also observed. Similar results were obtained in MTO1-silenced 143B cells (Fig. 2E).

Next we evaluated the capacity of oxidative phosphorylation in mutant and wild-type fibroblasts by determining the ATP levels after incubation of the cells in the presence of glucose or 2-deoxy-D-glucose with pyruvate (a treatment aimed to inhibit glycolysis without disturbing oxidative phosphorylation)^23^. The levels of total ATP in mutant cells grown with glucose were reduced by about 25% in comparison to wild-type cells (Fig. 2F, left), and by about 45% when glycolysis was inhibited (Fig. 2F, right). These data reveal an increased contribution of glycolysis to the ATP production in MTO1 fibroblasts and, accordingly, they suggest the existence of a cellular energetic reprogramming as an adaptive response to the MTO1 defect. An increase in the contribution of glycolysis to the energetic metabolism was also observed in MTO1-silenced cells (Fig. 2G).

Mutant fibroblasts exhibited mild changes in both membrane potential (Fig. S5A) and ROS production (Fig. S5B). No significant changes in these parameters were observed in MTO1-silenced cells (Fig. S5D and E). When the mutant fibroblasts were treated with hydrogen peroxide (H_2_O_2_), the ROS levels were not further increased in relation to the untreated cells (Fig. S5B), which suggests that an antioxidant system is already working in untreated MTO1 fibroblasts. Indeed, the mRNA levels of the antioxidant proteins TRX1, TRX2, PRDX3 and PRDX5 were found to be increased by 25–30% in these cells (Fig. S5C). A trend to the induction of these responses was observed in MTO1-silenced cells (Fig. S5F).

### MTO1 and GTPBP3 defects produce opposite effects on the HIF/PPARγ/UCP2/AMPK axis

AMPK is a metabolic sensor that plays a key role in maintaining cellular energy homeostasis. It is activated in response to an increase of the AMP/ATP ratio, stimulating ATP-producing catabolic pathways, like glycolysis and fatty acid oxidation, and inhibiting anabolic processes, including lipid biosynthesis^24^. Notably, research over the last decade has identified diverse molecular mechanisms that regulate the AMPK activity^24^. UCP2 is also a key regulator of energy metabolism that, by exporting intermediates of the TCA cycle, limits glucose oxidation, while promoting the oxidation of alternative substrates like glutamine and fatty acids^32–35^. Activation of AMPK has been involved in the up-regulation of UCP2 expression^36,37^, whereas UCP2 overexpression has been shown to increase signalling from AMPK^38^, although in both cases the underlying mechanisms remain unclear.

**Figure 3:**
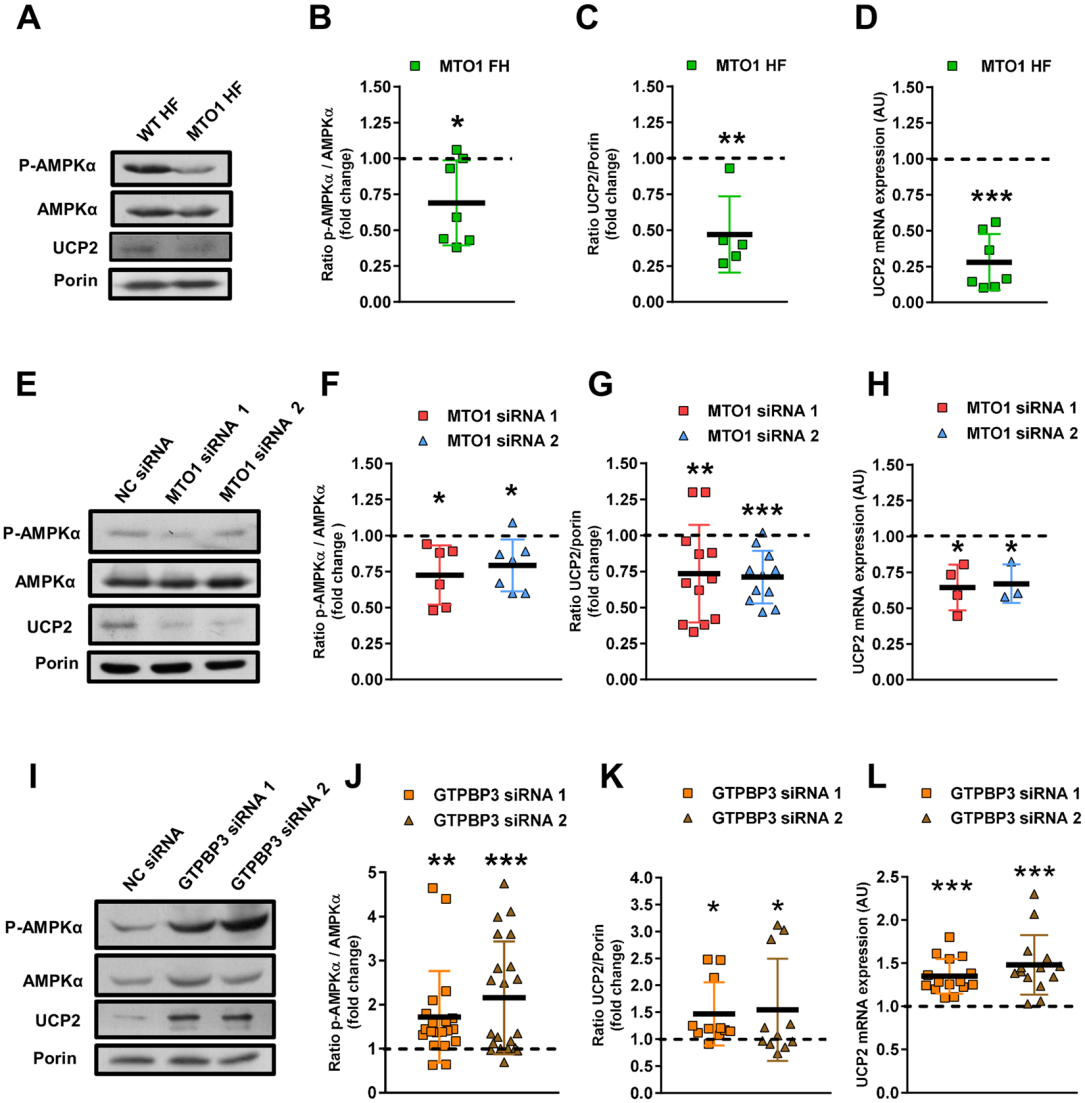
MTO1 and GTPBP3 defects produce opposite effects on the AMPK/UCP2 axis. **(A,E** and **I)** Representative immunoblots of phosphor-Thr172-AMPKα and UCP2 in wild-type (WT HF) and MTO1 (MTO1 HF) human fibroblasts (**A**), in MTO1 siRNA1-, MTO1 siRNA 2- and NC siRNA-transfected 143B cells (**E**), and in GTPBP3 siRNA1-, GTPBP3 siRNA 2- and NC siRNA-transfected 143B cells (**I**). AMPKα and porin were used as loading controls. Full-length western blots are included in supplementary information (Fig. S21). **(B,F** and **J)** Densitometric analysis of phospho-Thr172-AMPKα normalized to AMPKα and represented as fold change relative to control cells. **(C,G** and **K)** Densitometric analysis of UCP2 normalized to porin and represented as fold change relative to control cells. **(D,H** and **L)** qRT-PCR analysis of the *UCP2* mRNA expression in WT and MTO1 HF (**D**), in MTO1 siRNA1-, MTO1 siRNA 2- and NC siRNA-transfected 143B cells (**H**), and in GTPBP3 siRNA1-, GTPBP3 siRNA 2- and NC siRNA-transfected 143B cells (**L**). All data are the mean ± SD of at least three different experiments. Differences from control values were found to be statistically significant at *p < 0.05, **p < 0.01 and ***p < 0.001. NC: negative control.

We have reported that stable silencing of GTPBP3 in HEK-293 cells led to activation of AMPK and up-regulation of UCP2^18^. Therefore, we explored whether this response also occurs in MTO1 fibroblasts and in MTO1-depleted 143B cells. Unexpectedly, MTO1 fibroblasts showed a decrease in both the p-AMPKα/AMPKα ratio (Fig. 3A and B) as well as the UCP2 expression at mRNA (Fig. 3D) and protein levels (Fig. 3A and C). Similar results were obtained in MTO1 knocked-down 143B cells (Fig. 3E–H). In contrast, transient silencing of GTPBP3 in 143B cells (which reduced the GTPBP3 expression by about 50%, see Fig. S6) led to an increase in the p-AMPKα/AMPKα ratio (Fig. 3I and J), and UCP2 expression (Fig. 3K and L), as previously observed in HEK-293 cells^18^. These data suggest that the AMPK-UCP2 axis functions differently in MTO1- and GTPBP3-defective cells. Moreover, the finding that the cell response to the independent depletion of MTO1 and GTPBP3 was different in the same background, 143B cells, strongly suggests that one of these proteins has an additional role besides mt-tRNA modification.

**Figure 4:**
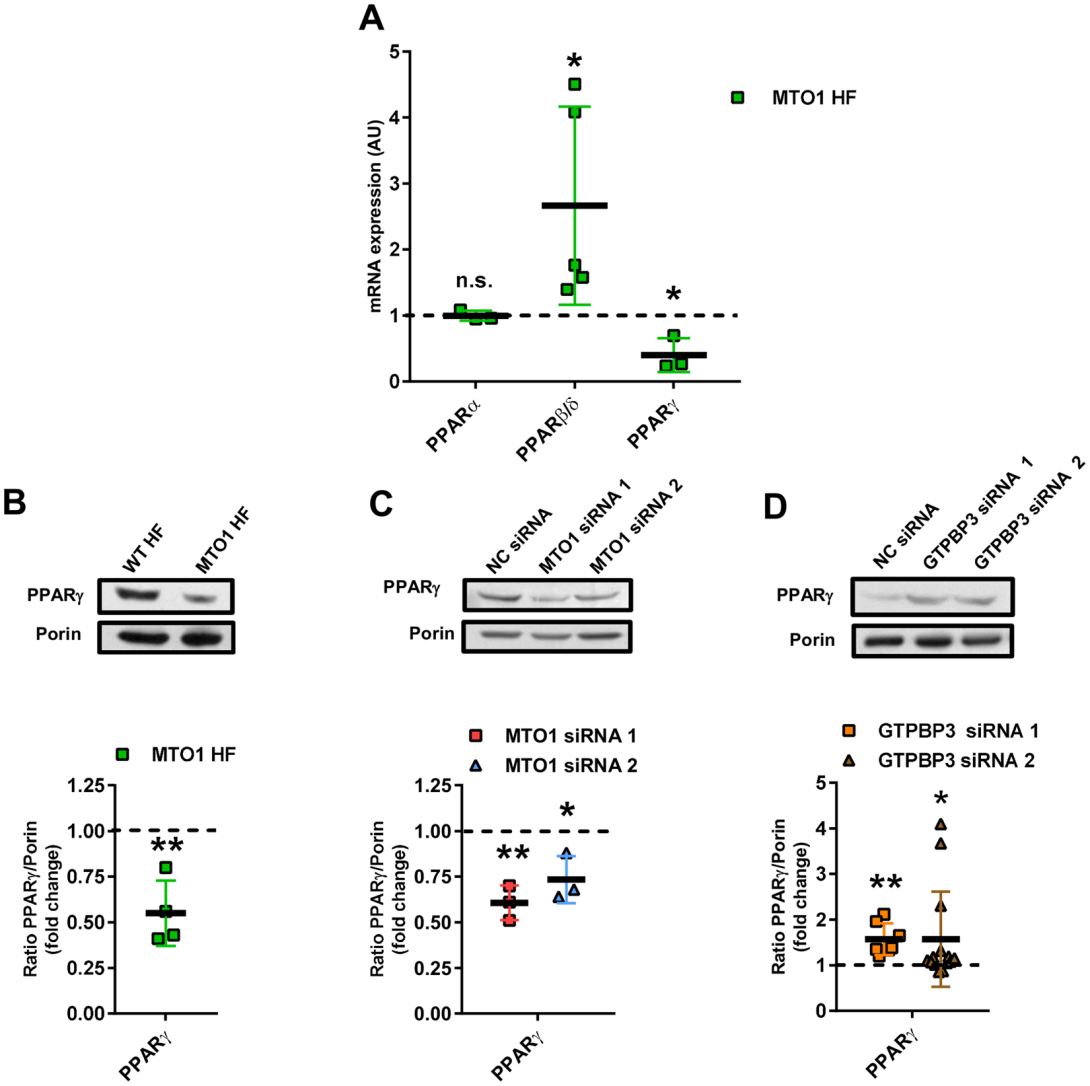
Expression of PPAR factors in MTO1 and GTPBP3 defective cells. (**A)** qRT-PCR analysis of the *PPARα, β/δ and γ* mRNA expression in MTO1 human fibroblasts (MTO1 HF). Data are represented as fold change respect to WT HF values (**B,C** and **D**) Representative immunoblots of PPARγ in WT and MTO1 HF (**B**), in MTO1 siRNA1-, MTO1 siRNA 2- and NC siRNA-transfected 143B cells (**C**), and in GTPBP3 siRNA1-, GTPBP3 siRNA 2- and NC siRNA-transfected 143B cells (**D**). The membranes were also probed with an antibody against porin, which was used as a loading control. Full-length western blots are included in supplementary information (Fig. S22). Scatter plots show the densitometric analysis of PPARγ normalized to porin and represented as fold change relative to control cells. All data are the mean ± SD of at least three different experiments. Differences from control values were found to be statistically significant at *p < 0.05 and **p < 0.01. n.s.: non-significant differences. NC: negative control.

**Figure 5:**
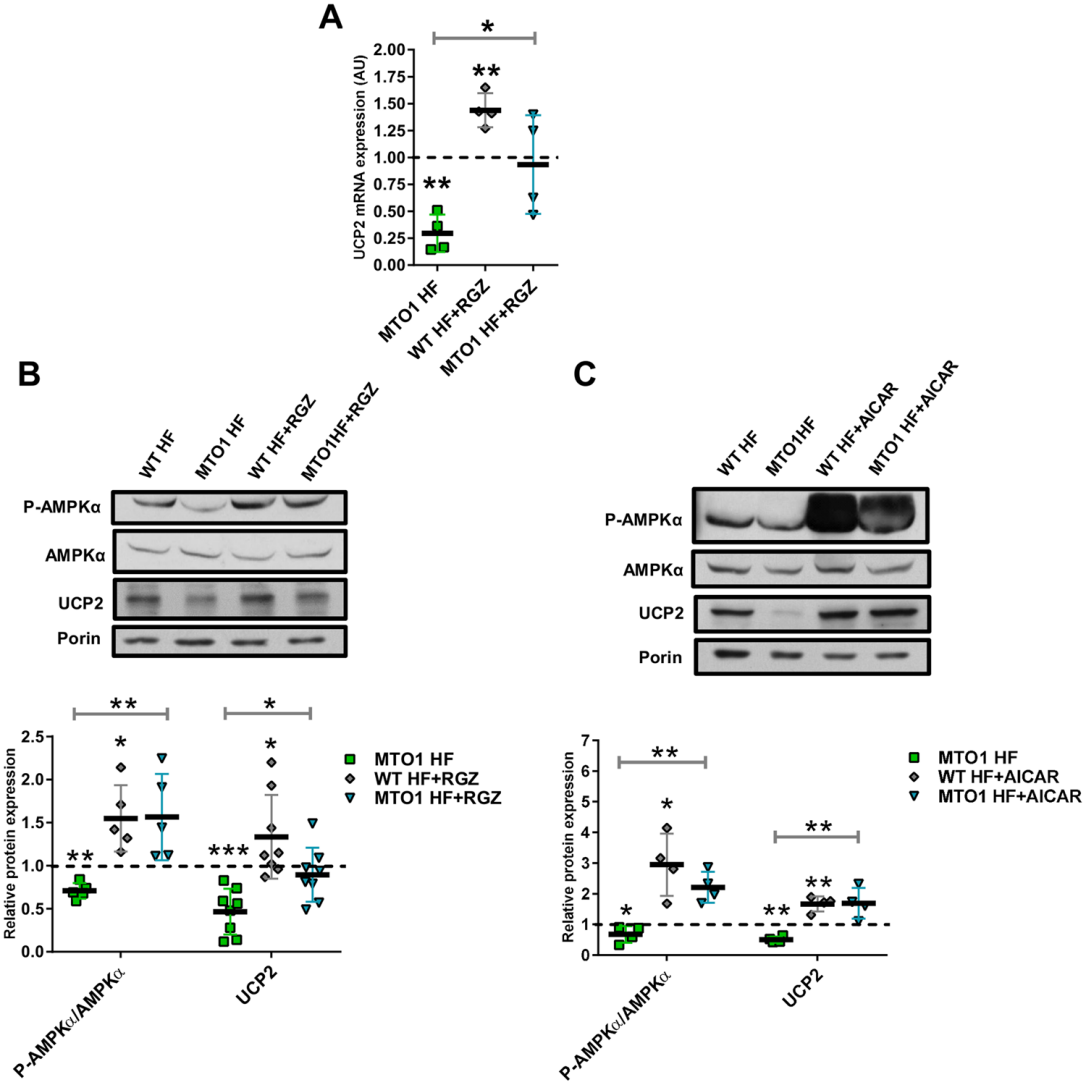
Regulation of UCP2 is AMPK- and PPARγ-dependent in MTO1 fibroblasts. **(A)** qRT-PCR analysis of the *UCP2* mRNA expression in MTO1 human fibroblasts (MTO1 HF), treated or not with 5 μM rosiglitazone (RGZ) for 1 h. Data are represented as fold change respect to WT HF values. **(B)** Representative immunoblots of phosphor-Thr172-AMPKα, AMPKα and UCP2 in WT HF and MTO1 HF, treated or not with 5 μM RGZ for 1 h. Porin and AMPKα were used as loading controls. Full-length western blots are included in supplementary information (Fig. S23). The scatter plot shows densitometric data for UCP2 normalized to porin and phosphor-Thr172-AMPKα normalized to AMPKα, and represented as fold change relative to WT HF. **(C)** Representative immunoblots of phosphor-Thr172-AMPKα, AMPKα and UCP2 in WT HF and MTO1 HF, treated or not with 1 mM AICAR for 1 h. Porin was used as a loading control. Full-length western blots are included in supplementary information (Fig. S23). The scatter plot shows densitometric data for UCP2 normalized to porin and phosphor-Thr172-AMPKα normalized to AMPKα, and represented as fold change relative to WT HF. All data are the mean ± SD of at least three different experiments. Differences from WT HF values were found to be statistically significant at *p < 0.05, **p < 0.01 and ***p < 0.001.

UCP2 is regulated at multiple levels^39^. Notwithstanding, the data showing that the UCP2 mRNA levels were reduced in MTO1 fibroblasts (Fig. 3D) and in MTO1-silenced cells (Fig. 3H), but increased in GTPBP3-silenced cells (Fig. 3L) suggest that UCP2 expression is controlled at transcriptional level in all these cells. Nuclear receptors PPARs (PPARα, PPARβ/δ, and PPARγ), which regulate lipid metabolism^40,41^, were selected as candidates in searching for UCP2 transcriptional regulators since previous studies have indicated that this hormone receptor family can regulate UCP2 expression via different mechanisms^39^. As shown in Fig. 4A, MTO1 fibroblasts exhibited an approximately 2-fold increase in the PPARβ/δ mRNA levels and a 2-fold decrease in the expression of PPARγ. We also found that the protein levels of PPARγ were reduced in MTO1 fibroblasts (Fig. 4B) and MTO1-silenced cells (Fig. 4C), but increased in GTPBP3-silenced cells (Fig. 4D), thus paralleling the mRNA expression pattern of UCP2 in each cell model (Fig. 3D,H and L). These data suggest that PPARγ regulates the transcriptional expression of UCP2 in the UCP2-AMPK axis that operates in both MTO1- and GTPBP3-defective cells although in a different manner. In fact, treatment of wild-type and MTO1 fibroblasts with rosiglitazone (RGZ), a PPARγ agonist, increased both the UCP2 expression (Fig. 5A and B) and the p-AMPK/AMPK ratio (Fig. 5B) with respect to untreated cells. It should be mentioned that RGZ has been shown to activate both PPARγ and AMPK, and that both activations are discrete events without any cross-talk^42^. Therefore, our data do not inform whether the increased expression of UCP2 produced by RGZ occurs through PPARγ or AMPK or both. The PPARγ protein levels were not affected by the RGZ treatment (Fig. S7) suggesting that PPARγ expression is not self-regulated, as previously reported^43^. We also found that treatment of wild-type and MTO1 fibroblasts with AICAR, an AMPK activator, led to an increase of the p-AMPKα/AMPK ratio and UCP2 protein levels (Fig. 5C). Notably, treatment of GTPBP3 stably-silenced cells with Compound C (an AMPK inhibitor that also inhibits respiration^44^) reduced the UCP2 mRNA levels in these cells^18^. Altogether these data highlight the importance of the PPARγ-UCP2-AMPK axis in the cell response to cope with the MTO1 or GTPBP3 deficit.

It is noteworthy that treatment of wild-type and MTO1 fibroblasts with a PPARβ/δ antagonist (GSK0660) did not affect the expression of UCP2 (Fig. S8), indicating that PPARβ/δ does not act as a regulator of UCP2 in these cells.

**Figure 6:**
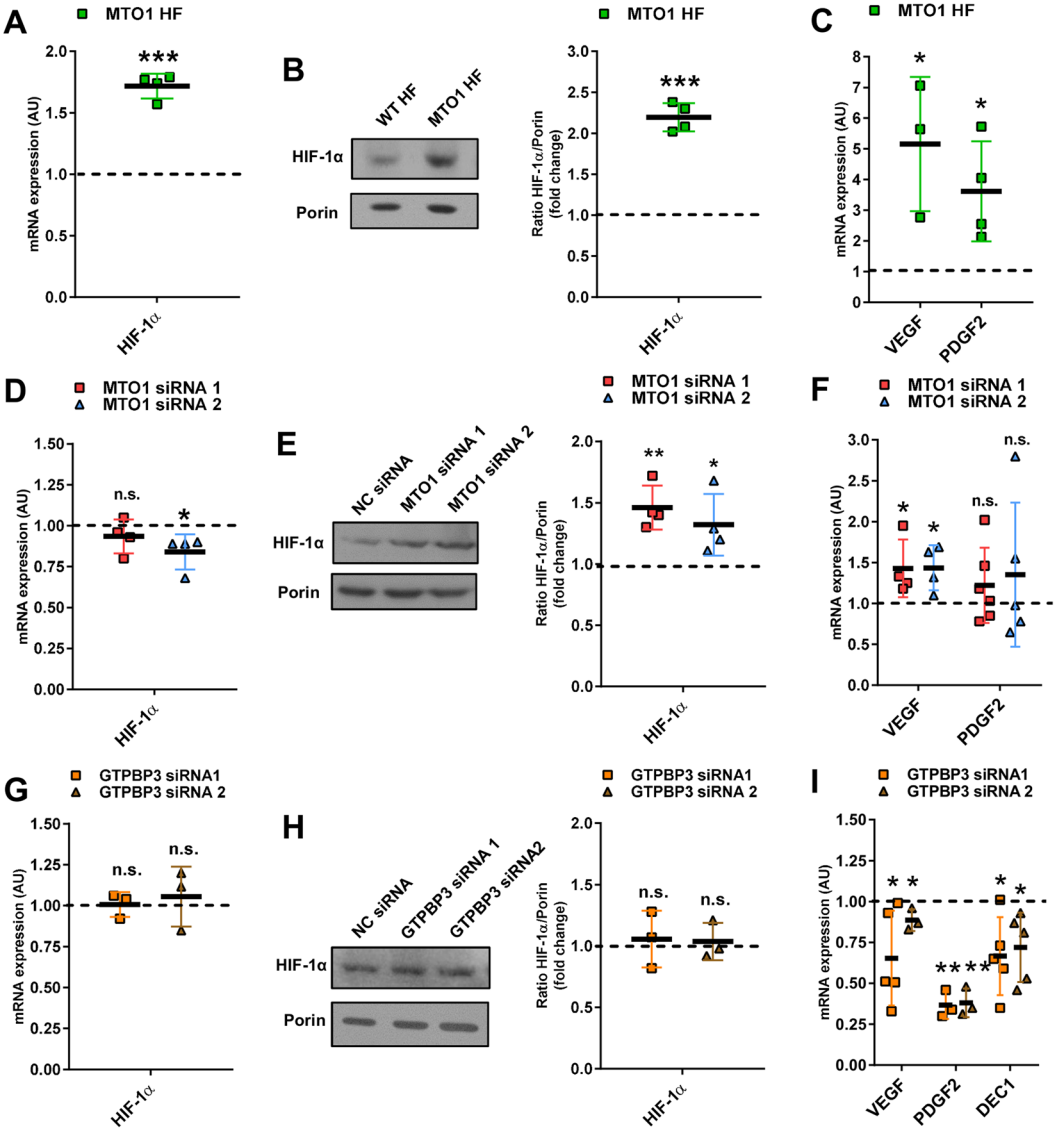
MTO1 and GTPBP3 defects produce opposite effects on HIF-1 signaling. (**A,D** and **G)** qRT-PCR analysis of the *HIF-1* mRNA expression in MTO1 HF (**A**), in MTO1 siRNA1- and MTO1 siRNA 2-transfected 143B cells (**D**), and in GTPBP3 siRNA1- and GTPBP3 siRNA 2-transfected 143B cells (**G**). **(B,E** and **H)** Representative immunoblots of HIF-1 in MTO1 HF (**B**), in MTO1 siRNA1- and MTO1 siRNA 2-transfected cells (**E**), and in GTPBP3 siRNA1- and GTPBP3 siRNA 2-transfected cells (**H**). Porin was used as a loading control. Full-length western blots are included in supplementary information (Fig. S24). The scatter plot shows densitometric data for HIF-1 normalized to porin, and represented as fold change relative to WT HF (**B**) or NC siRNA-transfected cells (**E** and **H**). **(C**,**F** and **I)** qRT-PCR analysis of mRNA expression of the HIF-1 target genes *vascular endothelial growth factor* (*VEGF*) and *platelet derived growth factor subunit 2* (*PDGF2*) in MTO1 HF (**C**), in MTO1 siRNA1- and MTO1 siRNA 2-transfected cells (**F**), and in GTPBP3 siRNA1- and GTPBP3 siRNA 2-transfected cells (**I**). All data are the mean ± SD of at least three different experiments and are represented as fold change respect to WT HF or NC siRNA-transfected cells. Differences from WT or NC values were found to be statistically significant at *p < 0.05, **p < 0.01 and ***p < 0.001. n.s: non-significant differences. NC: negative control.

Hypoxia-inducible factor (HIF) is a heterodimeric transcription factor that regulates glycolysis, cancer metabolism and cancer cell proliferation^45–47^. Under normal oxygen tension, the HIFα subunit is subject to oxygen-dependent prolyl hydroxylation by PHDs (prolyl-hydroxylase domain-containing enzymes), which promotes its degradation^46^. However, the prolyl-hydroxylase activity of PHDs can be inhibited by a number of intracellular factors, including ROS, nitric oxide, and TCA cycle intermediates like succinate and fumarate^46^. Activation of AMPK signalling in UCP2-overexpresing cells has been found to be associated with a downregulation of HIF expression^38^. Moreover, it has been recently shown that hypoxia decreased UCP2 via HIF-1-mediated suppression of PPARγ^48^. Considering that there are a growing number of mechanisms which allow for the activation of HIF-1 under normal oxygen conditions^49–52^, we asked whether HIF-1 is involved in regulation of PPARγ in MTO1- and GTPBP3-defective cells. We found an increase in the HIF-1α protein levels in both MTO1 fibroblasts and MTO1-depleted 143B cells (Fig. 6B and E), which was accompanied by an increase of the HIF-1α mRNA levels only in the case of MTO1 fibroblasts (Fig. 6A and D). Moreover, two canonical HIF-1 target genes, *VEGF* and *PDGF2*, were up-regulated in MTO1 fibroblasts and MTO1-silenced cells (a non-significant increase of the *PDGF2* mRNA levels was observed in MTO1-silenced cells) (Fig. 6C and F). Treatment of MTO1 fibroblasts with the HIF-1 inhibitor PX-478 upregulated the expression of UCP2 (Fig. S9A), reduced the PDGF2 expression (Fig. S9A), and led to an increase in the p-AMPK/AMPK ratio (Fig. S9B). Altogether these data suggest that HIF-1 is induced in the MTO1-defective cells and that it is a regulator of PPARγ and AMPK activity in these cells.

Remarkably, no changes in the HIF-1α expression were detected in GTPBP3-depleted 143B cells (Fig. 6G and H), although the mRNA levels of VEGF and PDGF2 were significantly decreased (Fig. 6I). A third HIF-1-responsive gene, *DEC1/Stra13*, whose product (DEC1) is recognized as a transcriptional repressor of PPARγ^53^ and SREBP-1C^54^, and as a negative regulator of the AMPK activity^55^, was then analyzed and found to be down-regulated in GTPBP3-depleted 143B cells (Figs 6I and S10A). Considering that the interaction of HIF-1 with the transcriptional coactivator p300/CBP is necessary for expression of HIF-1-responsive genes^46^, and that some proteins control the access of HIF-1 to p300/CBP^56,57^, it is possible that transactivation of basally expressed HIF-1 is inhibited in GTPBP3-depleted cells, which could facilitate the induction of PPARγ and UCP2, whereas suppressing the expression of VEGF, PDGF2 and DEC1. Notably, silencing of HIF-1 in 143B cells up-regulated the expression of PPARγ and UCP2, and increased the pAMPK/AMPK ratio (Fig. S9C).

Together our data are compatible with the idea that a HIF-PPARγ-UCP2-AMPK axis is involved in the phenotype of both MTO1- and GTPBP3-defective cells but operating in a different manner.

### MTO1 fibroblasts exhibit altered transcriptional expression of metabolism genes

**Figure 7:**
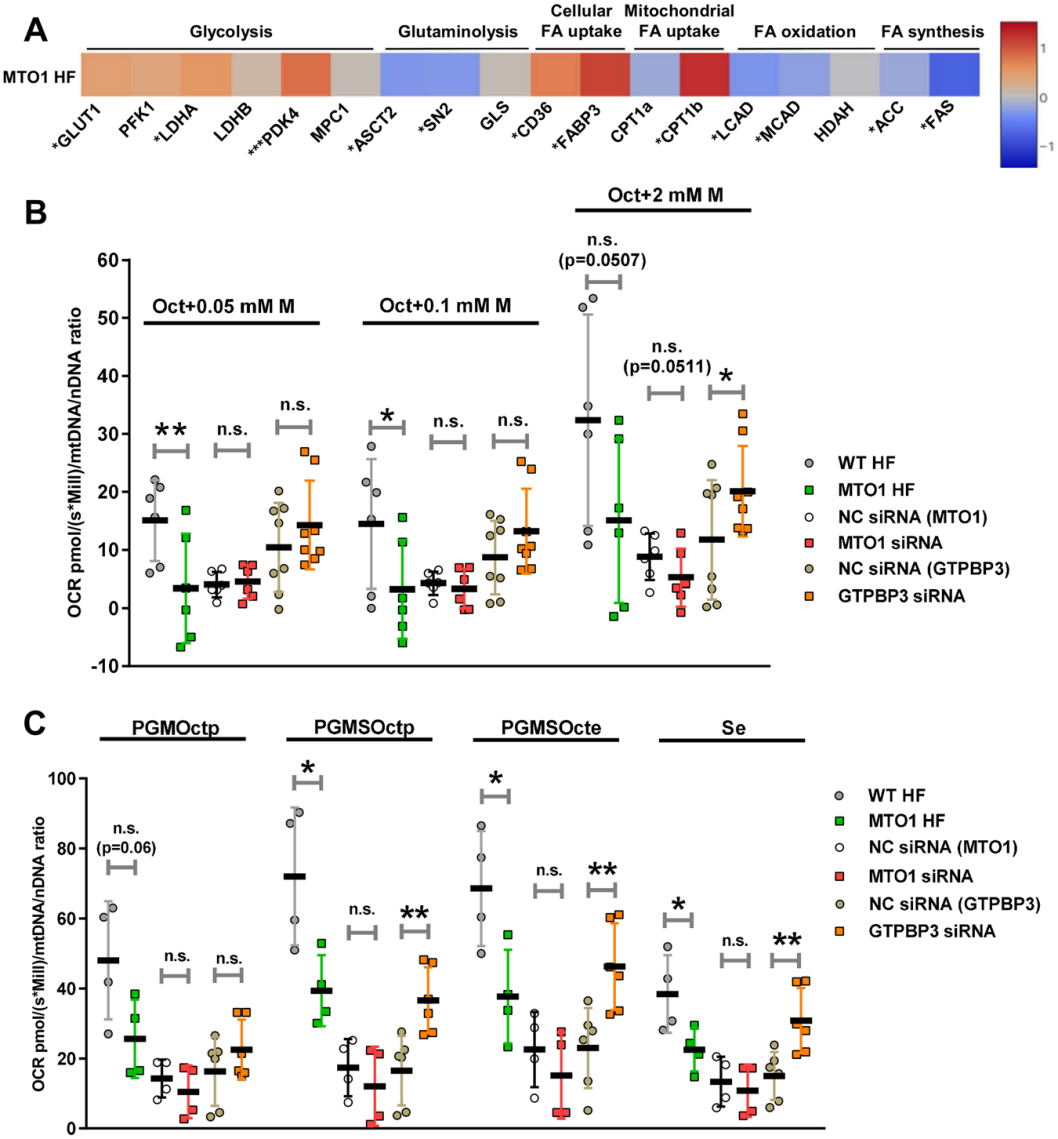
MTO1-defective cells exhibit altered expression of metabolism genes and differ from GTPBP3-defective cells in the bioenergetics profile. (**A**) qRT-PCR analysis of mRNA expression of genes related to glycolysis (*GLUT1: glucose transporter 1, PKF1: phosphofructokinase, LDHA* and *LDHB: lactate dehydrogenase A* and *B, respectively, PDK4: pyruvate dehydrogenase kinase 4* and *MPC1: mitochondrial pyruvate carrier 1*); glutaminolysis (*ASCT2: glutamine/amino acid transporter 2, SN2: glutamine/amino acid transporter system N*, and *GLS: glutaminase*); cellular fatty acid (FA) uptake (*FAT/CD36: fatty acid translocase*, and *FABP3: fatty acid binding protein 3*); mitochondrial fatty acid (FA) uptake (*CPT1a* and *CPT1b: carnitine palmitoyltransferase I a* and *b*); fatty acid oxidation (*LCAD: long-chain acyl-CoA dehydrogenase, MCAD:medium-chain acyl-CoA dehydrogenase*, and *HADH: hydroxyacyl-CoA dehydrogenase*); fatty acid synthesis (*ACC: Acetyl-CoA carboxylase* and *FAS: fatty acid synthase*), in MTO1 (MTO1 HF) human fibroblasts. Data are expressed as fold change respect to WT HF and shown in a heatmap. The colour and the corresponding value in log2 scale are plotted on the right. (**B**) Oxygen consumption rates of digitonin-permeabilized cells in the presence of 5 mM ADP, 0.2 mM octanoyl-carnitine (Oct) and malate (**M**), which was added at three different concentrations (0.05, 0.1 and 2 mM). (**C**) Oxygen consumption rates of digitonin-permeabilized cells in the presence of 5 mM ADP, 0.2 mM octanoyl-carnitine (Oct), 2 mM malate (**M**) and after sequential addition of substrates for Complex I (5 mM pyruvate (**P**) and 10 mM glutamate (**G**); PGMOctp) and Complex II (succinate (**S**); PGMSOctp), the uncoupler carbonyl cyanide-p-trifluoromethoxyphenylhydrazone (CCCP, stepwise titration in 0.05 μM increments; PGMSOcte), and the Complex I inhibitor (0.5 μM rotenone; Se). Oxygen consumption rates (OCRs), expressed as picomoles (pmol) per second (s) per million of cells (Mill), were normalized to the mitochondrial copy number (mtDNA/nDNA ratio) in each sample. p denotes phosphorylation of ADP + Pi to ATP. e denotes electron transfer system (ETS) capacity at optimum CCCP concentration (noncoupled respiration). Data represent the means ± SD from at least 3 independent determinations. Differences from wild-type (WT) or Negative Control (NC) values were found to be statistically significant at *p < 0.05, **p < 0.01 and ***p < 0.001. n.s.: non-significant differences.

Considering the key roles played by the components of the HIF-PPARγ-UCP2-AMPK axis in cellular energy homeostasis, we examined the mRNA expression of metabolism genes in MTO1 fibroblasts. The qRT-PCR analysis revealed increased mRNA levels of genes involved in glycolysis and fatty acid (FA) uptake, and decreased mRNA levels of genes involved in FA metabolism and glutaminolysis (Fig. 7A).

The increased expression of the glucose transporter 1 (GLUT1) and lactate dehydrogenase A (LDHA) may reflect an enhanced glycolysis (from glucose to pyruvate) in MTO1 fibroblasts (Fig. S11), which would be in agreement with the decrease in the ATP levels observed after incubation of the MTO1 fibroblasts with 2-deoxy-D-glucose and pyruvate (Fig. 2F). However, the increased expression of PDK4 in MTO1 fibroblasts and MTO1-silenced cells (Figs 7A, S12A and B) suggests that pyruvate oxidation is reduced in MTO1-defective cells since PDK4 is the enzyme responsible for the phosphorylation and inactivation of the pyruvate dehydrogenase (PDH). Thus, PDK4 induction could orchestrate an uncoupling between glycolysis and the TCA cycle (and, therefore, OXPHOS) in MTO1 fibroblasts (Fig. S11). Moreover, the increased mRNA levels of LDHA in MTO1 fibroblasts (Fig. 7A) suggests that lactate production is activated in these cells, likely to provide NAD+ for glycolysis to keep going. High glycolysis rates and uncoupled OXPHOS are possible causes of lactic acidosis^58^, which is a common symptom in most MTO1 patients^3,4,9^.

In MTO1 fibroblasts, we also observed a decreased expression of glutamine importers ASCT2 and SN2, and no significant change in the expression of GLS glutaminase (Fig. 7A). These data suggest that glutaminolysis is reduced in MTO1 fibroblasts.

Genes involved in the incorporation of fatty acids into the cell (CD36 and FABP3) and mitochondria (CPT1b) were found to be up-regulated in MTO1 fibroblasts, while expression of genes involved in fatty acid oxidation (LCAD and MCAD) and fatty acid synthesis (ACC and FAS) was found to be reduced (Figs 7A, S12C and D). Together these data suggest that fatty acid metabolism can be importantly perturbed in MTO1 fibroblasts (Fig. S11).

*GLUT1*, *LDHA* and other glycolytic genes are direct targets of HIF-1^45,47^. Thus, HIF-1 stabilization may contribute to increase glycolysis in MTO1 fibroblasts, which can be relevant considering that activity of AMPK, other stimulator of glycolysis, is decreased in these cells (Fig. 3A and B). *PDK4* and *CPT1b* are direct targets of PPARβ/δ, while the *ACC* and *FAS* genes, required for *de novo* lipogenesis, are regulated by the sterol regulatory element binding protein-1c (SREBP-1c) transcription factor. The expression of PPARβ/δ and SREBP-1c was found to be upregulated and downregulated, respectively, in MTO1 fibroblasts (Figs 4A and S13A), thus paralleling the expression of their targets (Fig. 7A). The regulatory role of PPARβ/δ on PDK4 and CPT1b genes was validated by treating MTO1 fibroblasts with a PPARβ/δ antagonist (GSK0660), which produced a decrease in the expression of both genes (Fig. S13B).

In brief, the transcriptional profile of MTO1 fibroblasts supports the proposal that the MTO1 defect promotes a drastic reprogramming of cell metabolism.

### MTO1- and GTPBP3-defective cells exhibit different fatty acid metabolism: lipid droplets accumulate in MTO1 fibroblasts

β-oxidation is a multistep oxidative process by which fatty acid (FA) molecules are broken down in mitochondria to generate acetyl-CoA, which enters into the TCA cycle, and NADH and FADH_2_, which are used, respectively, by complex I and the electron–transferring flavoprotein (ETF) complex for passing electrons to CoQ. To evaluate whether MTO1 and GTPBP3 defective cells have different ability to oxidize fatty acids, we determined OCR using malate and the active form of octanoate (octanoylcarnitine) as substrates^59^. Inclusion of malate brings about sustained oxidation of octanoylcarnitine by preventing accumulation of acetyl-CoA and replenishing matrix CoA^59^. We found that OCR was significantly reduced in MTO1 fibroblasts under most malate conditions, whereas it was increased in GTPBP3-silenced cells (Fig. 7B). A trend to decreased OCR was observed in MTO1-silenced cells (Fig. 7B). These data suggest that FA oxidation is impaired in MTO1-defective cells whereas it is favoured in GTPBP3-depleted cells, which is in agreement with the mRNA expression of MCAD and LCAD exhibited by each cell type (decreased in MTO1 fibroblasts, Fig. 7A, and increased in GTPBP3 stably-silenced cells,^18^). These data also fit with the role assigned to UCP2 in promoting FA oxidation^32,34^, as UCP2 levels are increased in GTPBP3-defective cells but are decreased in MTO1-defective cells (Fig. 3).

It is possible that the decreased OCR observed in MTO1 fibroblasts (Fig. 7B) was partially due to impairment of the OXPHOS system since reduced steady-state levels of complexes I and IV were detected in these cells (Fig. 2C). Therefore, we explored OCR of permeabilized MTO1- and GTPBP3-defective cells after sequential addition to the octanoylcarnitine-malate mix of: (1) substrates for complex I (pyruvate, glutamate and malate); (2) substrate for complex II (succinate); (3) the CCCP uncoupler; and (4) a complex I inhibitor (rotenone). As shown in Fig. 7C, MTO1 fibroblasts exhibited impaired OCR under most conditions, which supports the idea that impairment of the electron transport chain at several points in these cells prevents an efficient use of the provided substrates. In contrast, the GTPBP3-silenced cells were able to efficiently use complex II to compensate any putative impairment of complex I.

**Figure 8:**
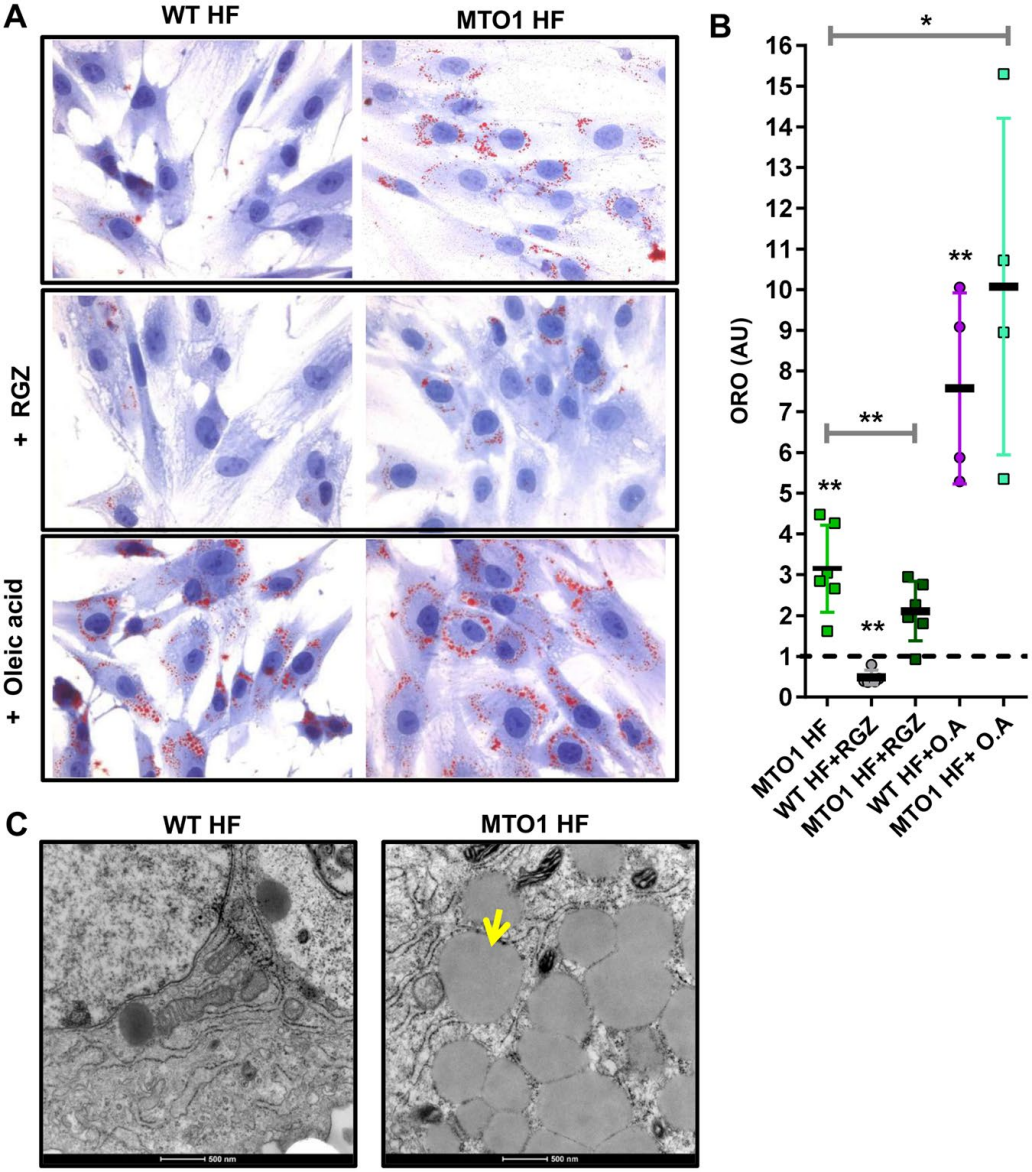
The MTO1 mutation p.Arg464Cys causes lipid accumulation in MTO1 fibroblasts. **(A)** Representative microscope pics of intracellular lipid droplets stained with Oil Red O (ORO) in wild-type (WT HF) and MTO1 (MTO1 HF) human fibroblasts under normal conditions (top panels), treated with 5 μM Rosiglitazone (RGZ) (middle panels), and exposed to 200 μM BSA-conjugated oleic acid (bottom panels). **(B)** Ratio of cells with lipid droplets (red) in relation to the total number of cells. Results are expressed as fold change relative to WT HF. Data represent the means ± SD from at least 3 independent determinations. Differences from WT HF values were found to be statistically significant at *p < 0.05 and **p < 0.01. **(C)** Representative electron micrographs (magnification x8200) of WT HF and MTO1 HF showing lipid droplets in MTO1 HF. The yellow arrow marks a lipid droplet. The scale bar corresponds to 500 nm. Pictures are representative from at least three independent experiments.

Given that OCR data did not provide clear evidence of reduced FA oxidation in MTO1 fibroblasts, we reasoned that the combination of defective fatty acid utilization and enhanced fatty acid uptake could trigger lipid accumulation in these cells (Fig. S11). In pathological states, the accumulation of lipid droplets is considered as a marker for increased content of toxic lipid metabolites and dysregulation of FA metabolism^60,61^. Therefore, we stained MTO1 and wild-type fibroblasts with Oil Red O dye, which allows selective detection of neutral lipids within cultured cells. MTO1 cells exhibited higher intracellular content of lipid droplets than wild-type (Fig. 8A (top panels) and B). Accumulation of lipid droplets was also detected by electron microscopy (Fig. 8C). Lipid content was reduced after treatment of fibroblasts with the PPARγ agonist rosiglitazone (RGZ) (Fig. 8A (middle panels) and B) or AICAR (Fig. S14), which suggests that activation of signalling from the PPARγ-UCP2-AMPK axis improves lipid handling in MTO1 fibroblasts. Notably, further increase of lipid content was observed after treatment of cells with oleic acid, a long-chain unsaturated fatty acid (Fig. 8A (bottom panels) and B). Altogether these data indicate that FA metabolism is deregulated in MTO1 fibroblasts.

## Discussion

Our results clearly indicate that the MTO1 p.Arg464Cys mutation or depletion of MTO1 protein levels leads to a reprogramming of cell metabolism mediated by the HIF-PPARγ-UCP2-AMPK axis and the contribution of PPARβ/δ and SREBP-1c. MTO1 fibroblasts exhibit HIF-1 activation, down regulation of PPARγ, UCP2 and SREBP-1c, up regulation of PPARβ/δ, and inactivation of AMPK. Most of these traits were also investigated in MTO1-silenced cells (143B background) and found to be similar, although usually less severe, to those of MTO1 fibroblasts. Due to the metabolic reprogramming, utilization of FA for the *de novo* synthesis of FA and for β-oxidation appears to be compromised, leading to accumulation of lipid droplets in MTO1 fibroblasts. Our data indicate that by targeting components of the HIF-PPARγ-UCP2-AMPK axis (e.g., using rosiglitazone or AICAR), it is possible to reverse the operating manner of the axis and, accordingly, lipid accumulation. We also demonstrate that depletion of the GTPBP3 protein in 143B cells produces the opposite effect to that observed after MTO1 depletion. GTPBP3-silenced cells showed functional inactivation of HIF-1, induction of UCP2 and PPARγ, and activation of AMPK, which results in efficient FA oxidation and increased OCR. Therefore, the MTO1 and GTPBP3 defects trigger a different cellular response in the same cell type (Fig. S15).

Our data on the ATP content of MTO1-defective cells grown in the presence of 2-deoxy-D-glucose with pyruvate indicate that glycolysis is increased in these cells. This increase could be mediated by the action of HIF since the expression of some glycolytic genes that are targets of HIF-1, like GLUT1 and LDHA^45,47^, is induced in MTO1 fibroblasts. Glycolysis also appeared to be increased in GTPBP3 stably-silenced HEK293 cells, as inferred from the induced expression of GLUT1 and LDHB^18^. However, in this case it could be mediated by the activation of AMPK since it has been shown that AMPK can promote GLUT1 and LDHB expression^62,63^. Both in MTO1-defective cells as well as in GTPBP3 stably-silenced cells, glycolysis appears to be uncoupled from OXPHOS although via different mechanisms: increased expression of PDK4 (the enzyme that prevents pyruvate oxidation by inactivating the pyruvate dehydrogenase complex) in MTO1-defective cells, and decreased expression of MPC1 (one of the two subunits that form the human mitochondrial pyruvate carrier) in GTPBP3 stably-silenced cells^18^. In both cases, the uncoupling is expected to result in lactic acidosis, a common clinical trait present in MTO1 and GTPBP3 patients. Uncoupling between glycolysis and OXPHOS has been shown to increase proton and lactate production in the heart, which can be detrimental to this organ^64^.

Although FAs are the principal energy substrate for the healthy adult hearts, cardiomyocytes can also use glucose and other energy sources including lactate, amino acids, and ketones^60,61,64^. The metabolic reprogramming observed in MTO1 fibroblasts involves a decrease of FA- and glucose-oxidation together with lipid accumulation, which could explain the relatively low level of AMPK phosphorylation observed in these cells as AMPK activity is inhibited by high levels of FA^24^. A scenario in cardiomyocytes similar to that observed in fibroblasts could contribute importantly to the heart failure in MTO1 patients.

GTPBP3-depleted cells exhibited increased OCR when respiration was sustained by either FA oxidation (via the ETF complex) or succinate (via complex II). These data are in agreement with our previous report indicating that GTPBP3 stably-silenced cells show induced expression of genes involved in FA oxidation and increased complex II activity^18^. Notably, the ATPase activity of complex V was also found to be increased, which could contribute to the reduced ATP levels observed in GTPBP3-depleted cells^18^. This feature merits to be further investigated in patient cells since reduced ATP levels may be detrimental for organs with high energy demand like the heart^65^.

The finding that the cell response activated by the MTO1 deficiency was different from that triggered by the GTPBP3 deficiency is striking since both proteins are involved in the same mt-tRNA modification process. Here, we present evidence that the pathological MTO1 change p.Arg464Cys or low levels of MTO1 cause hypomodification of substrate mt-tRNAs. GTPBP3 silencing also affected the modification status of these mt-tRNAs^18^. Therefore, both proteins conserve the tRNA modification activity of their bacterial and yeast homologs^66,67^. However, the different cell response triggered by the deficit of each human protein indicates that one of them has an additional function besides mt-tRNA modification. A few data from the literature support this proposal: null mutations in the bacterial and yeast *MTO1* homologous genes produce a slightly more severe phenotype than null mutations in the *GTPBP3* homologs^68,69^. These data suggest that the MTO1 homologs have an additional function. Interestingly, recent data suggest that MTO1 could be involved in mitoribosome assembly^8^. A second function of MTO1 could explain for the differences in the expression of the HIF-PPARγ-UCP2-AMPK axis found between MTO1- and GTPBP3-defective cells since retrograde signalling triggered by MTO1 impairment would be different from that triggered by GTPBP3 impairment. This second function of MTO1 would also be affected by the clinical mutation p.Arg464Cys. Further work is needed to clarify the expression pattern of the HIF-PPARγ-UCP2-AMPK axis in cells from patients carrying other MTO1 mutations.

Our study suggests that despite deficiencies in either MTO1 or GTPBP3 cause hypertrophic cardiomyopathy with lactic acidosis, the underlying metabolic signalling in patient cells may be different (see Supplementary Discussion). The retrograde signals that are activated in each case remain unclear but they are probably related to the specific stress caused by each defect on mitochondrial translation and OXPHOS function. Overall, this work reveals the importance of the HIF-PPARγ-UCP2-AMPK axis in the metabolic reprogramming of the MTO1- and GTPBP3-defective cells, highlighting the modulation of this axis as a potential therapeutic strategy.

## Methods

### Materials

Rosiglitazone (5-[[4-[2-(Methyl-2-pyridinylamino)ethoxy]phenyl]methyl]-2,4-thiazolidinedione) and AICAR (5-Aminoimidazole-4-carboxamide 1-β-D-ribofuranoside) were purchased from Sigma and Tocris, respectively. GSK0660(3-(((2-Methoxy-4-(phenylamino)phenyl)amino]sulfonyl)-2-thiophenecarboxylic acid methyl ester) was purchased from abcam. PX-478 was purchased from Selleckchem. Oligonucleotides (Table S3) were purchased from Sigma and Qiagen.

### Bacterial strains, plasmids and microbiological media

*E. coli* strains used in this work were MG1655 (wild-type) and IC5241 (*mnmG::Tn10*)^70^. IC5241 strain was transformed with plasmids pBAD22 (empty plasmid carrying the arabinose-inducible promoter P_BAD_), pIC1180 (pBAD22 expressing MnmG fused to the N-terminal end of a Flag-epitope (FLAG-MnmG)) and pIC1750 (pBAD22 expressing FLAG-MnmG with p.Arg427Cys mutation). PIC1750 plasmid was obtained from pIC1180 by site-directed oligonucleotide mutagenesis (QuickChange^TM^, Stratagene). Plasmid pCR3.1 was used for cloning human MTO1 cDNA (pCR3.1-MTO1) and GTPBP3 cDNA (pCR3.1-GTPBP3) containing a six-histidine tail (His tag) at the 3′ end. pCR3.1-MTO1mut (carrying p.Arg464Cys mutation) was obtained from pCR3.1-MTO1 by site-directed oligonucleotide mutagenesis. A list of the oligonucleotides handled in this work is provided in Table S3. All constructs were verified by DNA sequencing. *E. coli* DH5α cells were used for overproduction and purification of pBAD22, pIC1180, pIC1750, pCR3.1, pCR3.1-MTO1, pCR3.1-MTO1mut, pCR3.1-GTPBP3 plasmids. Bacterial strains were grown in LBT (Luria-Bertani-broth containing 40 μg/ml thymine). Antibiotics were added when required (ampicillin at 100 μg/ml and tetracyclin at 12.5 μg/μl).

### Cell culture and plasmid transfections

Fibroblasts cells were obtained from a patient carrying a homozygous mutation in the *MTO1* gene (NM_0121233: c.1392 C>T; p.Arg464Cys)^9^. The c.1392 C>T; p.Arg464Cys mutation is equivalent to the c.1510 C>T; pArg504Cys mutation in the reference sequence (NM_001123226) used in^9^. Written informed consent was obtained from the patient’s parents, and the study was approved by the Ethics Committee of Hospital 12 de Octubre (Madrid, Spain). All methods involving human samples were performed in accordance with the relevant guidelines and regulations. Control fibroblasts were obtained from the Coriell Institute for Medical Research (GM01582). Wild-type and patient fibroblasts cells were cultured in MEM medium supplemented with 10% Fetal Bovine Serum (FBS), 1% penicillin–streptomycin, 1 mM essential and non-essential amino acids, 1% vitamin and 0,5% sodium hydroxide. Human osteosarcoma 143B cells (ATCC CRL-8303) were cultured in high glucose Dulbecco’s modified Eagle medium (Gibco) containing 10% FBS, 1 mM sodium pyruvate, 100 U/ml penicillin, 100 μg/ml streptomycin, 2 mM glutamine and 1 mM non-essential amino acids. Human HeLa cells (ATCC CCL-2) were grown in full medium: Minimum Essential Medium (MEM) (Sigma) supplemented with 10% heat-inactivated fetal bovine serum, 100 U/ml penicillin and 100 μg/ml streptomycin. All cell cultures were kept at 37 °C in a humidified atmosphere with 5% CO2.

143B cells and HeLa cells were used for transient transfections. 143B cells were seeded at 200,000 cells/well in 6 well-plate, 1,000,000 cells/100 mm dish or 1,500,000 cells/75 cm^2^ flask 1 day before transfection. 143B cells were transfected with two different Invitrogen Stealth siRNAs targeting *MTO1* (MTO1 siRNA1 (HSS119344) and MTO1 siRNA 2 (HSS119345)), *GTPBP3* (GTPBP3 siRNA 1 (HSS189171) and GTPBP3 siRNA 2 (HSS150080)) and *HIF-1* (HIF-1 siRNA 1 (HSS104774) and HIF-1 siRNA 2 (HSS179231)) genes or with negative control (NC) siRNAs (High GC duplex (12935400) and Medium GC Duplex (12935300)) at the 50 nM final concentration, using Lipofectamine 2000 reagent (Invitrogen) according to the manufacturer’s instructions. The cells were processed after two days of transfection. Silencing of *MTO1* and *GTPBP3* was periodically determined by quantitative PCR and/or western blot analysis (Figs S2A and B, S6A and B, and S9C). HeLa cells were seeded at 0.75–1 × 10^5^ on coverslips in 24-well plates. After 24 h, cells were transfected with pCR3.1 or pCR3.1 derivative plasmids expressing human MTO1 (pCR3.1-MTO1), mutant MTO1 (pCR3.1-MTO1mut) or GTPBP3 (pCR3.1-GTPBP3) using Lipofectamine 2000 reagent (Invitrogen), according to the manufacturer’s instructions. The cells were processed for immunofluorescence as described below after two days of transfection.

### Isolation of bulk tRNA from *E. coli* and reverse-phase HPLC analysis of nucleosides

Total tRNA purification and analysis of nucleosides by reverse-phase HPLC were performed as described previously^67,71^. HPLC analysis was monitored at 314 nm to achieve optimal adsorption of thiolated nucleosides. Nucleosides were identified according to their UV spectra and by comparison with appropriate controls.

### RNA isolation and qRT-PCR

Total and small RNA were isolated using TRIzol reagent (Invitrogen) and NucleoSpin miRNA kit (Macherey-Nagel), respectively, following the manufacturer’s instructions. For mRNA quantification, one-step qRT-PCRs were performed in an Applied Biosystems Step-One Real-Time PCR System. Total RNA (50–150 ng) was reverse-transcribed and amplified by qPCR in 20 μl of total volume reaction containing specific primers (Sigma), Power SYBR Green PCR Master Mix, MultiScribe Reverse Transcriptase, and RNase Inhibitor (all from Applied Biosystems), according to the manufacturer’s instructions. Relative quantitation of mRNA levels was calculated using the comparative Ct method. *ACTB* was used as endogenous control. A list of the primers used in this work is provided in Table S3.

### In vitro cleavage reaction of human total small RNA and Northern Blotting analysis

The cleavage reaction mixtures (1 μg of purified human total small RNA, 2.5 μg/ml recombinant angiogenin (ANG), 30 mM HEPES pH 7.4, 30 mM NaCl and 0.01% bovine serum albumin) were incubated at 37 °C for the indicated times and quenched by adding 5 μl of Gel Loading Buffer II (Life Technologies). Cleavage products from human RNA samples were resolved in 15% denaturating polyacrilamide gels with 7 M urea, and then transferred to positively charged nylon membranes (Mannheim Boehringer). Pre-hybridization and hybridization were performed with Dig Easy Hyb (Roche) according to the manufacturer’s instructions. mt-tRNA^Val^ and mt-RNA^Lys^ were detected with specific DIG-labeled synthetic oligodeoxynucleotides (Table S3). Quantification of non-radioactive signals was performed with ImageQuant TL v8.1 (GE Healthcare Life Sciences).

### Flow cytometry studies

Cells were detached at 37 °C with trypsin-EDTA and resuspended in culture media. Mitochondrial membrane potential was measured by incubating cells in suspension (10^6^ cells/ml) with 100 nM MitoTracker Red CMXRos for 30 min at 37 °C, and the emitted fluorescence (620 ± 20 nm band-pass filter) was recorded^72^. For ROS analysis, cells were treated or not with 0.3 mM H_2_O_2_ for 2 h and the media replaced by fresh media with 5 μM hydroethidine or 5 μM dihydrorhodamine 123 for 30 min at 37 °C. Then, cells were washed twice with phosphate buffered saline (PBS), trypsinized, and the emitted fluorescences, red (filter as above) or green (525 ± 20 nm band-pass filter) for hydroethidine or dihydrorhodamine 123, respectively, were measured^73^. For all the measurements, 10,000 cells were analysed and collected using a Cytomics FC 500 flow cytometer (Beckman Coulter).

### Measurement of intracellular ATP

The amount of ATP content was measured using an ATP bioluminescence assay kit (HSII; Roche), according to the manufacturer’s instructions. Luminescence was determined using the Spectra Max M5 (Molecular Devices).

### Blue-Native PAGE and Western Blot

BN-PAGE was performed similarly as described in^13^. Samples containing 15 μg of protein were separated on 3–12% Bis-Tris Novex NativePAGE gel (Life Technologies). The relative level of the assembled respiratory complexes I-IV was assessed by Western blot with commercially antibodies: mouse monoclonal anti-NDUFB8 antibody (sc-65237, Santa Cruz Biotechnology), mouse monoclonal anti-SDHA antibody (A11142, Molecular Probes), mouse monoclonal anti-Complex III subunit Core 1 antibody (459140, Invitrogen) and rabbit polyclonal anti-COXIV (4850, Cell signaling). Complex V was detected with a rabbit polyclonal antibody^74^.

For Western blots in eukaryotic cells, cells were processed as described in^18^. For immunodetection, the following antibodies were used: rabbit polyclonal anti MTO1 (15650-1-AP, Protein Tech), rabbit polyclonal anti-porin (ab15895, Abcam), rabbit polyoclonal anti-phosphoAMPKα (Thr172) (2531, Cell Signaling), rabbit polyclonal anti-AMPKα (2532, Cell Signaling), rabbit polyclonal anti-phosphoACC (Ser 79) (3661, Cell Signaling), rabbit polyclonal anti-ACC (3662, Cell Signaling), rabbit polyclonal anti-PDK4 (Q16654, ABGENT), rabbit monoclonal anti-CLPP (ab124822, Abcam), rabbit polyclonal anti-LONP1 (NBP1-81734, Novus Biologicals) and mouse polyclonal anti-AFG3L2 (ab68023, Abcam), rabbit polyclonal anti-ND6 (sc20667, Santa Cruz Biotechnology), rabbit polyclonal anti-COXI (459600, Invitrogen), mouse monoclonal anti-UCP2 (sc-390189, Santa Cruz Biotechnology), mouse monoclonal anti-PPARγ (sc-7273,Santa Cruz Biotechnology), mouse monoclonal anti-HIF1 (sc-13515), mouse monoclonal anti-DEC1 (sc-101023) and mouse monoclonal anti-Fas (sc-48357, Santa Cruz Biotechnology). Anti-GTPBP3 was purified from GTPBP3-His-inoculated rabbit serum^18^. The anti-goat (A5420), anti-rabbit (A6154) and anti-mouse (A4416) IgG-horseradish peroxidase-conjugated secondary antibodies were obtained from Sigma. Protein bands were quantified by densitometric analysis with an Image Quant ECL (GE Healthcare).

We determined the steady-state levels of the mnmG protein in the *E. coli* strains MG1655, IC5241 and IC5241 carrying pBAD22, pIC1180 or pIC1750 plasmids. Briefly, strains were grown in LBT (Luria-Bertani broth containing 40 μg/ml thymine) until OD_600_ reached 0.4. Then, the culture was divided into two equal parts, 0.2% L-arabinose (inducer of the AraC-PBAD system) was added to one of them, and incubation continued until OD_600_ reached 1. Fifty μg of total proteins were loaded in each well. For immunodetection of mnmG and GroEL, we used the following antibodies: anti-MnmG purified from MnmG-inoculated rabbit serum^70^ and mouse monoclonal anti-GroEL (ADI-SPS-870, Enzo).

### High Resolution Respirometry in Intact Cells using Oxygraph-2K (Oroboros)

Oxygen consumption rate (OCR) in fibroblasts and 143B cells was measured using a high-resolution respirometer (Oxygraph-2 k, Oroboros Instruments, Innsbruck, Austria), as described in^75^. In brief, 80% confluent cells were detached at 37 °C with trypsin-EDTA and resuspended in fresh growth media at concentrations 250,000 cells/mL for fibroblasts and 1,000,000 cells/mL for 143B cells. Each cell type was simultaneously analyzed in two 2 mL-Oxygraph chambers. A real-time measurement of the oxygen consumption rate (OCR) was performed at 37 C° in each chamber at basal conditions and after sequential addition of inhibitors for the different mitochondrial respiratory complexes: oligomycin (2.5 μg/ml) to inhibit complex V (to assess non-mitochondrial respiratory capacity or leak rate), carbonyl cyanide-p-trifluoromethoxyphenylhydrazone (CCCP) uncoupler with stepwise titration in 2.5 to 1.5 μM increments (to assess maximal electron transport system respiratory capacity rate), rotenone (0.5 μM) to inhibit complex I, and antimycin A (2.5 uM) to inhibit complex III. Data was analyzed using DatLab7 (Oroboros, Austria) software. Oxygen consumption rates (OCRs) were normalized to the mitochondrial copy number in each sample.

Oxygen consumption rate in permeabilized fibroblasts and 143B cells under ADP excess (state III) was also measured using the high-resolution respirometer. Fibroblasts and 143B cells were detached at 37 °C with trypsin-EDTA, washed once with phosphate buffered saline (PBS), once with respiration media MiR05 (110 mM sucrose, 60 mM potassium-lactobionate, 0.5 mM EGTA, 3 mM MgCl_2_·6H_2_O, 20 mM taurine, 10 mM KH_2_PO_4_, 20 mM HEPES adjusted to pH 7.1 with KOH at 37 °C; and 1 g/L BSA) and then resuspended in MiR05 media at concentrations 250,000 cells/mL for fibroblasts and 1,000,000 cells/mL for 143B cells. A real-time measurement of the oxygen consumption rate (OCR) was simultaneously performed in the two 2 mL-Oxygraph chambers for each cell type. Cells were first permeabilized using 10 μg digitonin and then substrates and inhibitors were sequentially added as follows: 5 mM ADP, 0.2 mM octanoyl-carnitine, 0.05 mM malate, 0.1 mM malate, 2 mM malate, 10 μM cytochrome c, 5 mM pyruvate, 10 mM glutamate, 10 mM succinate, stepwise titration of CCCP uncoupler in 0.05 μM increment as needed, 0.5 μM rotenone, 2.5 μM antimycin A. Mitochondrial membrane integrity was verified by the addition of cytochrome c (10 μM; the changes observed in OCR were always lower than 10%). Data was analyzed using DatLab7 (Oroboros, Austria) software. Oxygen consumption rates (OCRs), expressed as picomoles (pmol) per second (s) per million of cells (Mill), were normalized to the mitochondrial copy number in each sample.

### Mitochondrial DNA copy number quantification

mt-DNA copy numbers were obtained by real-time PCR as previously described^76^.

### Oil Red O staining and quantification

Lipid accumulation in fibroblasts cells was visualized by staining with Oil Red O (lipid soluble dye). The cells were washed twice with phosphate buffered saline (PBS), fixed with 4% paraformaldehyde (PFA) for 30 min, washed twice with distilled water (dH_2_O), incubated with 60% isopropanol for 5 min and stained with 60% Oil Red O solution for 15 min. After the staining, cells were washed with dH_2_O until excess stain was no longer apparent, then incubated with Hematoxylin (nuclei dye) for 1 min and washed with dH_2_O as needed. The lipid content of stained cells was visualized by microscopy (Philips CM10 transmission electron microscope); lipid droplets appear red and nuclei appear blue. Thirty fields were randomly selected from each sample. The number of cells with lipid droplets (red) (n) and the total number of nuclei (N) were counted for each field. Mean of the ratios (n/N) of all fields was calculated for each sample and, then, expressed as fold change relative to wild-type.

### Electron microscopy

Cells were seeded on Lab-Tek chamber slides (Nunc), washed and fixed with 3% glutaraldehyde. Then, they were post-fixed in 1% osmium tetroxide for 1 h, rinsed, dehydrated, incubated for 2 h with 2% of uranyl acetate and embedded in Araldite (Sigma-Aldrich). Ultrathin sections were cut, stained with lead citrate, and examined under a Philips CM10 transmission electron microscope.

### Fluorescence microscopy

HeLa cells were cultured on coverslips in 24-well plates. After transient transfections, cells were rinsed with PBS, fixed with 4% paraformaldehyde–PBS for 20 min at RT, washed with PBS, permeabilized with 0.3% Triton X-100 in PBS for 15 min and washed again with PBS. Then, cells were quenched in 100 mM NH4Cl, 150 mM glycine in PBS for 10 min, washed with PBS, and blocked with a solution containing 2% BSA, 0,05% Triton X-100 in PBS for 30 min at RT. Then, cells were incubated with 1:300-diluted anti-6 × His (631212, clontech) and anti-HSP60 (ab46798, Abcam) antibodies in blocking solution overnight at 4 C°. Upon washing with blocking solution, bound antibodies were subsequently detected by incubation, as appropriate, with 1:300-diluted AlexaFluor 594-conjugated anti-rabbit (11012, Invitrogen) and AlexaFluor 488-conjugated anti-mouse (A11001, Invitrogen) secondary antibodies in blocking solution for 1 h at 37 °C. Nuclei were counterstained with 0.1 μg/ml DAPI nuclear blue dye (Roche Diagnostics). Slides were mounted in FluorSave reagent (Calbiochem-Merck4Biosciences) and images were obtained with Apotome-equipped Axio Observer Z1 microscope (Carl Zeiss AG).

### Statistical analysis

The statistical analyses were performed using Graph Pad Prism 5. Student’s t-test was used in all comparisons of data. The statistically significant differences between the means were indicated by asterisks (*p < 0.05, **p < 0.01 or ***p < 0.001), and non-significant differences by n.s.

## Electronic supplementary material

Supplementary Information
