# Supplementary material for: Defects in the mitochondrial-tRNA modification enzymes MTO1 and GTPBP3 promote different metabolic reprogramming through a HIF-PPARγ-UCP2-AMPK axis
Source: Sci Rep. 2018 Jan 18;8:1163. doi: 10.1038/s41598-018-19587-5 (PMC5773609; doi:10.1038/s41598-018-19587-5)
Supplement: Supplementary file 1 — Supplementary Information [file 41598_2018_19587_MOESM1_ESM.pdf]

Supplementary Information

Defects in the mitochondrial-tRNA modification enzymes MTO1 and GTPBP3 promote different metabolic reprogramming through a HIF-PPARγ-UCP2-AMPK axis

Rachid Boutoual[1,2], Salvador Meseguer[1,*], Magda Villarroya[1], Elena Martín-Hernández[3], Mohammed Errami[2], Miguel A. Martín[4], Marta Casado[5], M.-Eugenia Armengod[1, 6,*]

[1]RNA Modification and Mitochondrial Diseases Laboratory, Centro de Investigación Príncipe Felipe (CIPF), Valencia 46012, Spain

[2]Faculty of Sciences, Abdelmalek Essaadi University, Tetouan BP.2121, Morocco

[3]Unidad de Enfermedades Mitocondriales y Enfermedades Metabólicas Hereditarias, Departamento de Pediatría, Hospital 12 de Octubre, Madrid 28041, Spain.

[4]Mitochondrial and Neuromuscular Disorders Laboratory, Hospital Universitario 12 de Octubre, Madrid 28041, and Centro de Investigación Biomédica en Red de Enfermedades Raras (CIBERER) nodo U723, Madrid 28029

[5]Instituto de Biomedicina de Valencia, IBV-CSIC, Valencia 46010, Spain, and Centro de Investigación Biomédica en Red de Enfermedades Hepáticas y Digestivas (CIBERehd), and Centro de Investigación Biomédica en Red de Enfermedades Cardiovasculares (CIBERcv), Madrid 28029, Spain.

[6]Centro de Investigación Biomédica en Red de Enfermedades Raras (CIBERER) node 721, Madrid 28029

***Corresponding authors** (smeseguer@cipf.es; marmengod@cipf.es)

Corresponding author for communication purposes with the journal staff:

M.-Eugenia Armengod; E-mail: marmengod@cipf.es; Phone: +34-963289680; Fax: +34-963289701

INDEX **PAGES**

INDEX	PAGES
Supplementary Discussion	S1-S3
Supplementary Figures S1 to S16	S4-S19
Supplementary Figures S17 to S24 (full-length blots of Figs 1-8)	S20-S27
Supplementary Tables S1 to S3	S28-S31
Supplementary References	S32-S33

SUPPLEMENTARY DISCUSSION

A working model of the signaling pathways operating in MTO1- and GTPBP3-defective cells

There are a growing number of mechanisms which allow for the activation of HIF-1 under normal oxygen conditions[1-4]. We propose that the MTO1 deficiency activates the expression of HIF-1 by a still unknown mechanism (Fig. S16). HIF-1 could promote both down-regulation of PPARγ and SREBP1c as well as AMPK inactivation through the action of DEC1/Stra13, as this protein has been shown to repress the *PPARγ2* promoter[5] and *SREBP-1c* transcription[6], and to negatively regulate AMPK activity via LKB1[7]. In fact, we observed an increase of DEC1 protein levels in MTO1-depleted cells (Fig. S10B). Down-regulation of PPARγ through HIF-1 activation could lead to down-regulation of UCP2 in MTO1-defective cells (as several works support that PPARγ is involved in UCP2 regulation; see for example[8,9]) and, accordingly, affect mitochondrial metabolism. Notably, HIF-1 has been shown to enhance glycolysis through transactivation of genes encoding glucose transporters (e.g., GLUT1) and glycolytic enzymes (e.g., LDHA), and to regulate mitochondrial metabolism through several mechanisms, including induction of *PDK4* expression and mechanisms affecting assembly and activity of respiratory complexes[10-12]. Moreover, HIF-1 reprograms lipid metabolism by inducing lipid droplet accumulation, stimulating fatty acid (FA) uptake (e.g. CD36), and inhibiting mitochondrial FA oxidation by means of several mechanisms, including suppression of *MCAD* and *LCAD* expression through inhibition of the c-MYC→PGC-1β axis[11,13]. Our transcriptional study of metabolism genes in MTO1 fibroblasts show that some HIF-1-responsive genes are induced in these cells (Fig. 7A).

In GTPBP3-depleted cells, we have observed an increase in protein levels of PPARγ (Fig. 4D) and UCP2 (Fig. 3I), but no change in the HIF-1α protein levels (Fig. 6H) despite three HIF-1-responsive genes (VEGF, PDGF2 and DEC1) are down-regulated in GTPBP3-depleted cells (Fig. 6I and Fig. S10A). Considering that the interaction of HIF-1 with the transcriptional

coactivator p300/CBP is necessary for the expression of the HIF-1-responsive genes[14], and that some proteins (e.g., CITED2, CITED4 and p35srj) control the access of HIF-1 to p300/CBP[15,16], we suspect that transactivation of basally expressed HIF-1 is inhibited by some p300/CBP binding protein in the GTPBP3-depleted cells. It has been proposed that p35srj may regulate HIF-1 transactivation by controlling access of HIF-1 to p300/CBP, and may keep a significant portion of p300/CBP available for interaction with other transcription factors[15]. Therefore, p35srj (or other protein) could carry out this role in GTPBP3-depleted cells. Interestingly, SREBP-1c has been reported to be acetylated at K289 and K309 by p300/CBP under certain conditions, which increases its stability[17]. Therefore, PPARγ could be activated through SREBP-1c (or other pathways) in GTPBP3-depleted cells and be responsible for induction of UCP2. Our data indicate that GTPBP3 silenced 143B cells exhibited increased OCR when respiration was sustained by either FA oxidation (via the EFT complex) or succinate (via complex II) (Fig. 7B and 7C). Therefore, it is intriguing why the ATPase activity of complex V was found to be increased in GTPBP3 stably-silenced HEK293 cells[18]. We have proposed that a reverse operation of complex V could be aimed at preventing a drastic drop of the membrane potential due to UCP2 activity[18]. Although the role of UCP2 as an uncoupling protein is controversial, future research should investigate whether the ATPase activity of complex V is also increased in 143B cells after knocking down GTPBP3 expression and, if so, whether there is a relationship between such an increase and the UCP2 induction.

Currently, the retrograde signals produced by the MTO1 and GTPBP3 deficiencies are unclear, but they could be related to the stress caused by each defect on mitochondrial translation and OXPHOS function. The different cell responses to each deficiency observed in this work strongly suggest that one of the two proteins, likely MTO1, has an additional function besides mt-tRNA modification, which could also be involved in mitochondrial translation. If so, the translational stress and, consequently, the resulting retrograde signaling could be different between GTPBP3- and MTO1-defective cells. It has been reported that the activation of cell responses to an impairment of mitochondrial

translation precedes respiratory chain deficiency[19]. The initial signal for this activation seems to be a significant buildup of unfolded and/or unassembled OXPHOS subunits leading to impairment of mitochondrial proteostasis, which generates a nuclear response. We speculate that the unfolded or unassembled OXPHOS subunits may be different in GTPBP3- and MTO1-deficient, which could produce different retrograde signaling and different modulation of the HIF-PPARγ-UCP2-AMPK axis. In addition, the MTO1 and GTPBP3 defects could also affect differently the OXPHOS function and, thus, the homeostasis of metabolites such as ROS, Ca^{2+}, ADP/ATP, and NAD/NADH, which modulate retrograde signaling pathways[20]. In fact, we have found that the MTO1-defect is associated with decreased complex I and IV levels (Figure 2D), while stable knock-down of GTPBP3 decreased only complex I levels[18], a features that could be related to the different ability of the two type of defective cells to use reduced equivalent coming from several sources (Figure 7, B and C). The effect of the GTPBP3 and MTO1 defects on the OXPHOS complexes can be a result of both the role played by each protein in mitochondrial translation and the nuclear response to each defect. In this respect, it should be mentioned that we have reported a decreased expression of the NDUFAF3 and NDUFAF4 complex I assembly factors in the GTPBP3-depleted cells, which could be partially responsible for the decrease in the complex I levels exhibited by these cells[18].

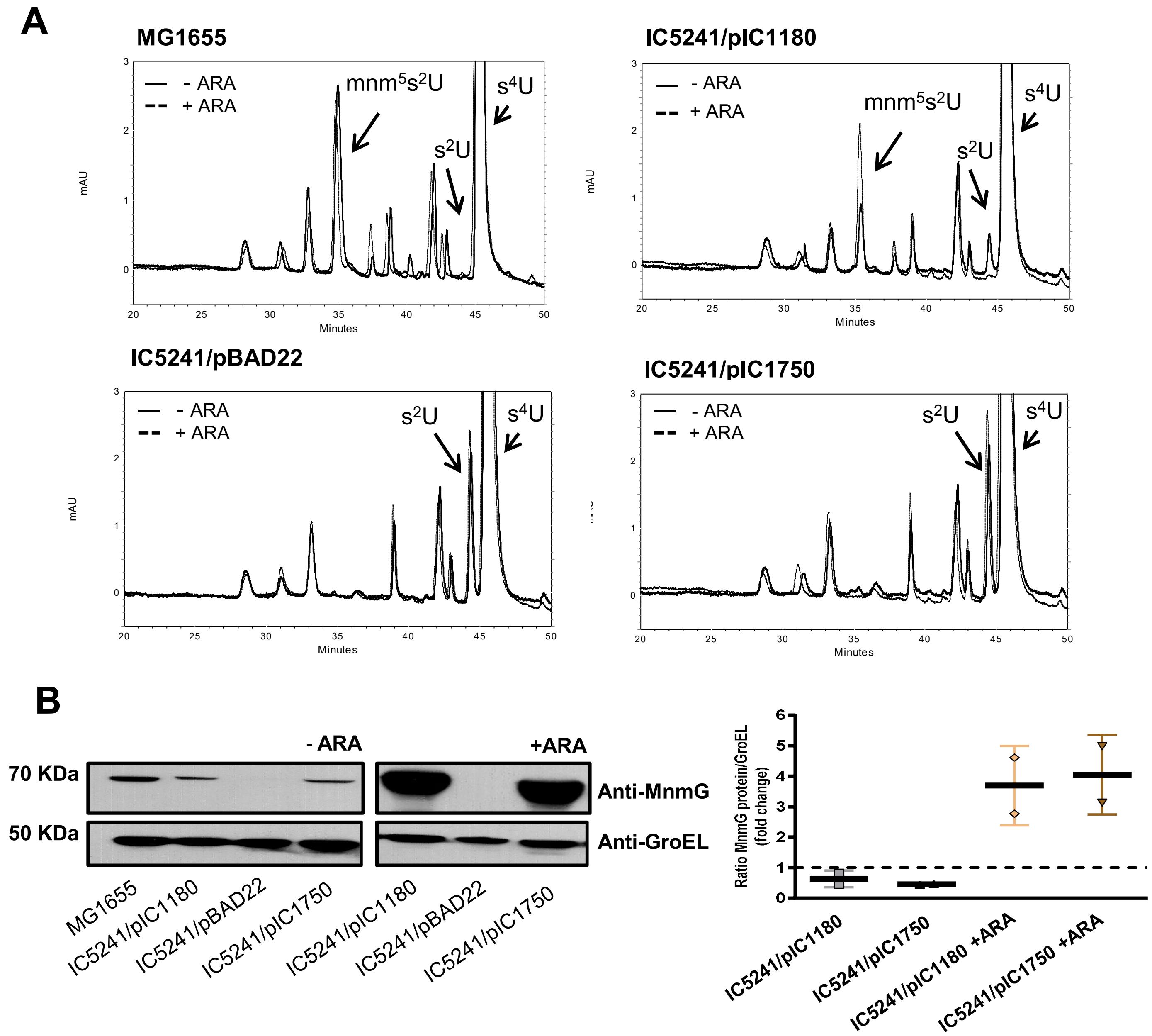

Figure S1. The clinical mutation p.Arg464Cys abrogates the tRNA modification activity of the *E. coli* MnmG protein. (A) HPLC analysis of the nucleoside composition of total tRNA extracted from a wild-type strain (MG1655) or a null *mnmG* mutant strain (IC5241) carrying either pBAD22 (IC5241/pBAD22) or its derivative plasmids, which express, under the control of the arabinose inducible promoter P_{BAD}, the wild type (IC5241/pIC1180) or mutant (IC5241/pIC1750) MnmG protein. The strains were grown in LBT with (+) or without (-) 0.2% ARA (L-arabinose) at 37C° for 2.5 h before being processed. The nucleosides were monitored at 314 nm to maximize the detection of thiolated nucleosides. The relevant nucleosides at position 34 (mnm^5s^2U and s^2U) and position 8 (s^4U) of tRNAs are indicated with arrows. AU, absorbance units. The HPLC analysis indicates that, both in the presence or absence of the inducer arabinose, the mutant MnmG protein expressed by pIC1750 was unable to modify *E. coli* tRNAs. **(B)** Representative immunoblots of MnmG in the indicated strains grown in the absence (-ARA) or presence (+ARA) as indicated above. Fifty μg of total proteins were loaded in each well. Size of the molecular weight marker is in kDa. The membranes were also probed with an antibody against GroEL, which was used as a loading control. The scatter plot shows the densitometric analysis of MnmG normalized to GroEL and represented as fold change relative to the data from MG1655. Data represent the means ± SD from 2 independent determinations.

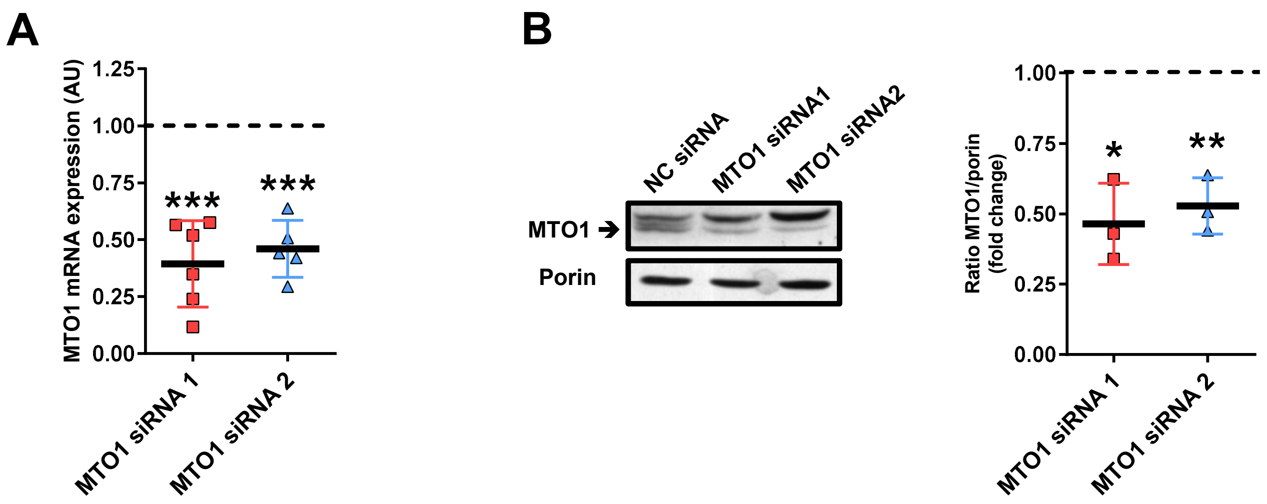

Figure S2. Transient silencing of MTO1 in osteosarcoma 143B cells. (A) qRT-PCR analysis of *MTO1* mRNA expression in MTO1 siRNA1- and MTO1 siRNA 2-transfected cells. **(B)** Western blot analysis of MTO1 expression in MTO1 siRNA1-, MTO1 siRNA 2- and NC (negative control) siRNA-transfected cells. The membranes were also probed with an antibody against porin, which was used as a loading control. The scatter plot shows the densitometric analysis of MTO1 normalized to porin. All data are the mean ± SD of at least three independent biological replicates and are expressed as fold change respect to the NC values. Differences from NC values were found to be statistically significant at *p<0.05, **p<0.01 and ***p<0.001.

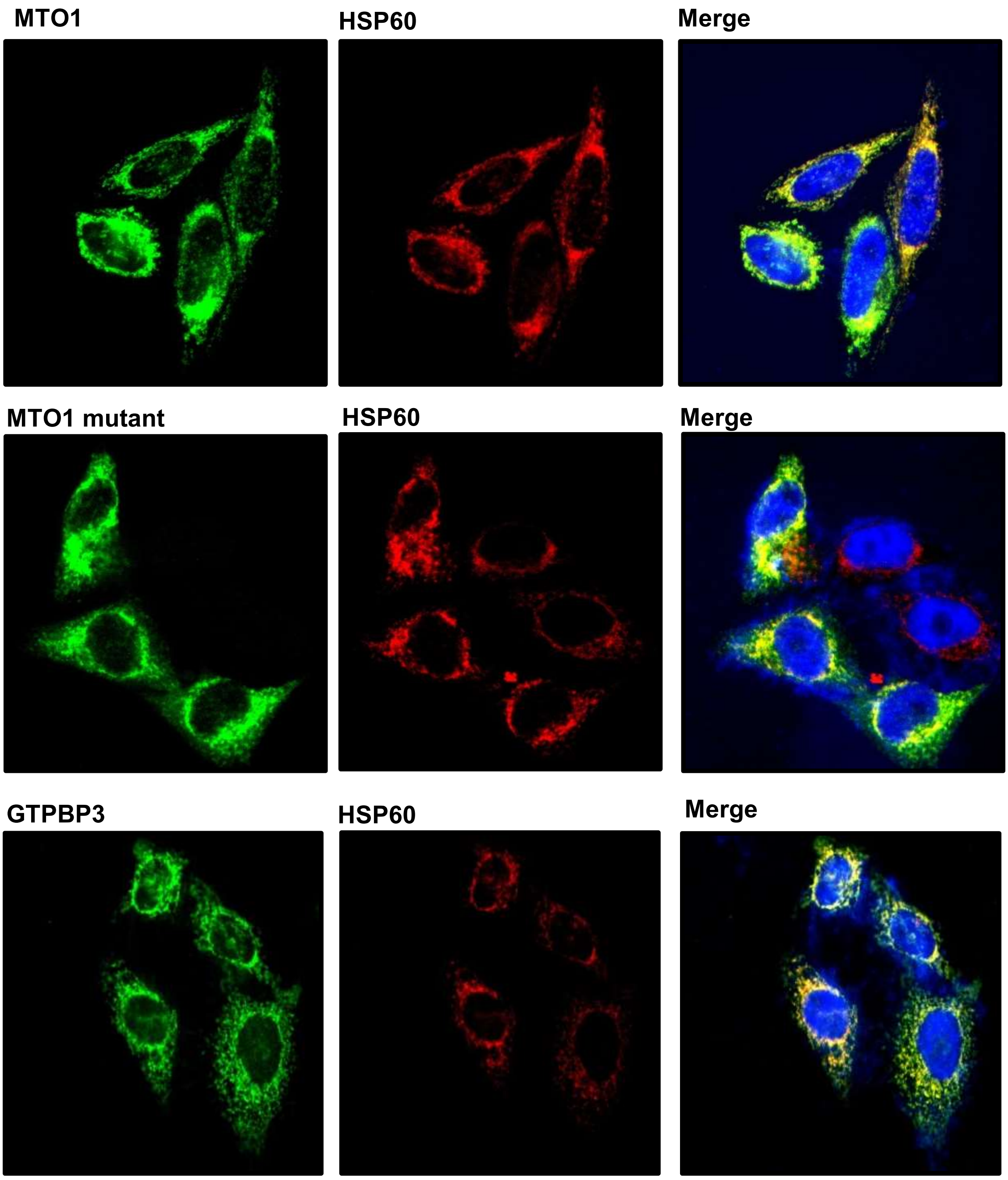

Figure S3. p.Arg464Cys mutation does not alter the mitochondrial localization of MTO1. HeLa cell lines were transiently transfected with pCR3.1, pCR3.1-MTO1, pCR3.1-MTO1mut and pCR3.1-GTPBP3. Intracellular distribution of MTO1, MTO1mut and GTPBP3 proteins was revealed by the reaction with monoclonal primary antibody against 6×His and the use of specific Alexa Fluor-488 (green fluorescence). The expression of HSP60 (Mitochondrial protein) in the same cells was assessed by the application of monoclonal antibody against HSP60 and the use of specific Alexa Fluor-594 (red fluorescence). Right panels show merged images for HSP60 dye and endogenously expressed protein MTO1, MTO1mut and GTPBP3 in HeLa cells. Yellow color shows colocalization of HS60 with MTO1, MTO1mut and GTPBP3. Nuclei (Blue) were visualized by DAPI staining. These images are representative of 3 separate experiments.

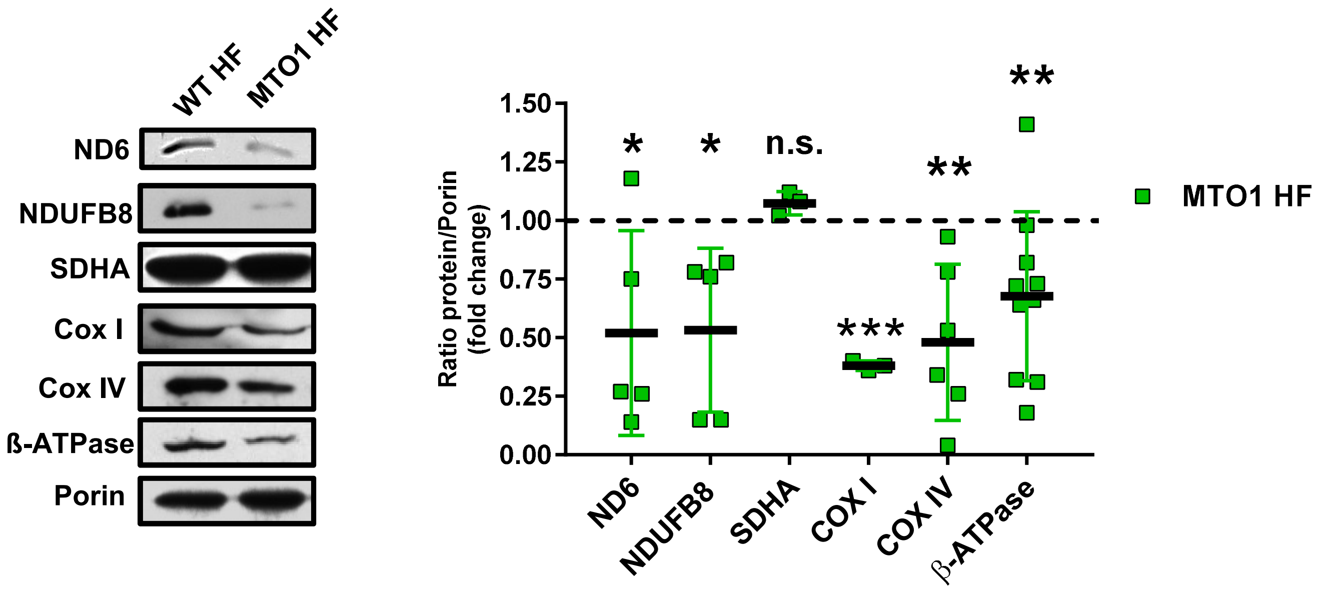

Figure S4. Reduced steady-state levels of OXPHOS subunits in MTO1 fibroblasts. Representative immunoblots of the OXPHOS subunits ND6 and NDUFB8 (Complex I), SDHA (Complex II), COXI and COXIV (Complex IV) and β-subunit (Complex V) in WT HF and MTO1 HF. The membranes were also probed with an antibody against porin, which was used as a loading control. The scatter plot shows the densitometric analysis of OXPHOS subunits normalized to porin and represented as fold change relative to WT. Data represent the means ± SD from at least 3 independent determinations. Differences from WT values were found to be statistically significant at *p<0.05, **p<0.01 and ***p<0.001. n.s: non-significant differences.

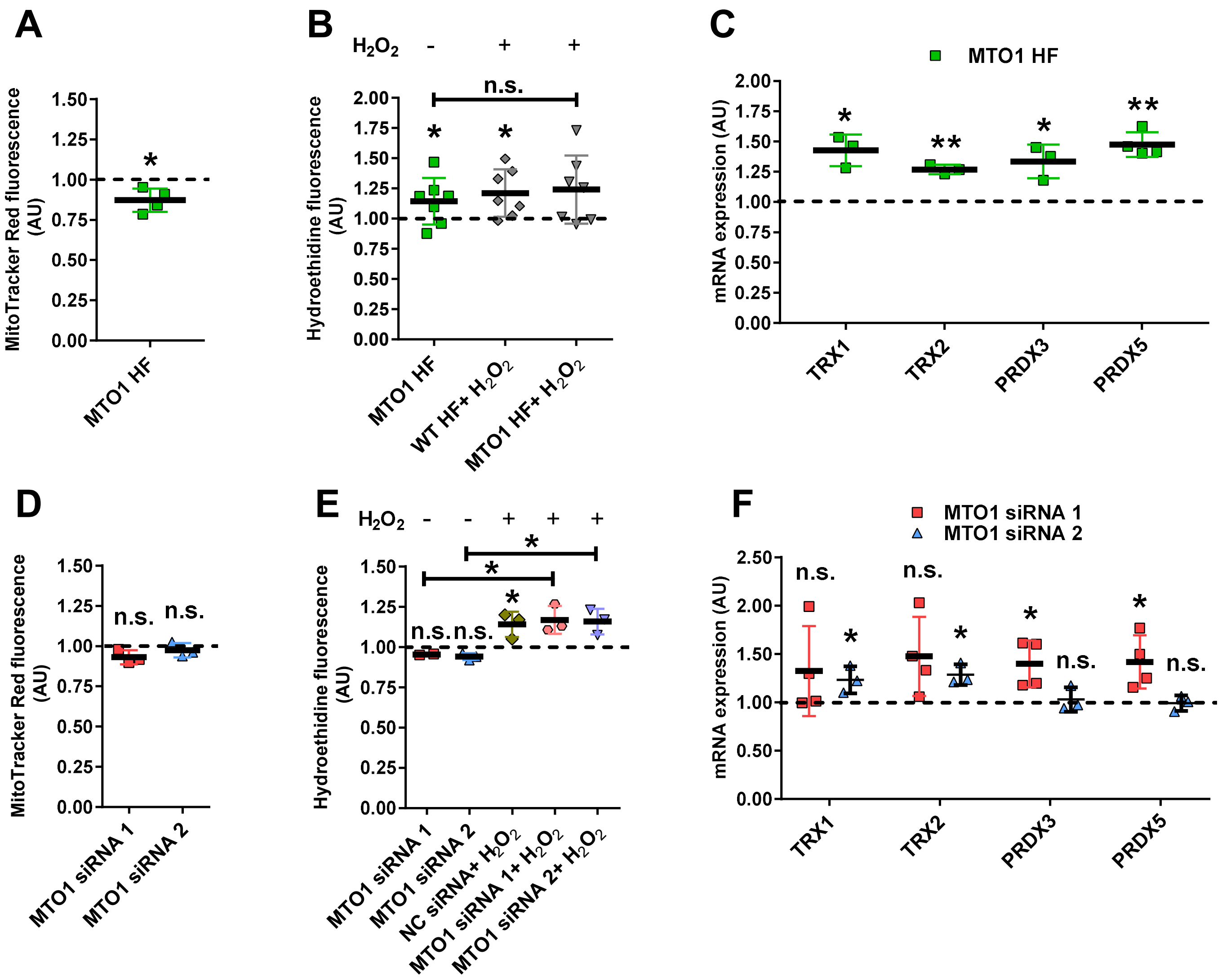

Figure S5. Effect of MTO1 deficiency on membrane potential, ROS production and antioxidant response. (A and D) Determination of mitochondrial membrane potential in MTO1 human fibroblasts (MTO1 HF) **(A)**, and MTO1-silenced 143B cells **(D)**. **(B and E)** Determination of ROS in MTO1 HF **(B)** and MTO1-silenced 143B cells treated (+) or not (-) for 2 h with 0.3 mM H2O2 **(E)**. **(C and F)** qRT-PCR analysis of Thioredoxin-1 (THRX1), Thioredoxin-2 (THRX2), Peroxiredoxin-3 (PRDX3) and Peroxiredoxin-5 (PRDX5) mRNA expression in MTO1 HF **(C)**, and MTO1-silenced 143B cells **(F)**. All data are the mean ± SD of at least three different experiments and are expressed as fold change respect to the WT fibroblasts values (A-C) or to NC (negative control siRNA-transfected cells) values (D-F). Differences from control values were found to be statistically significant at *p<0.05, **p<0.01. n.s.: non-significant differences.

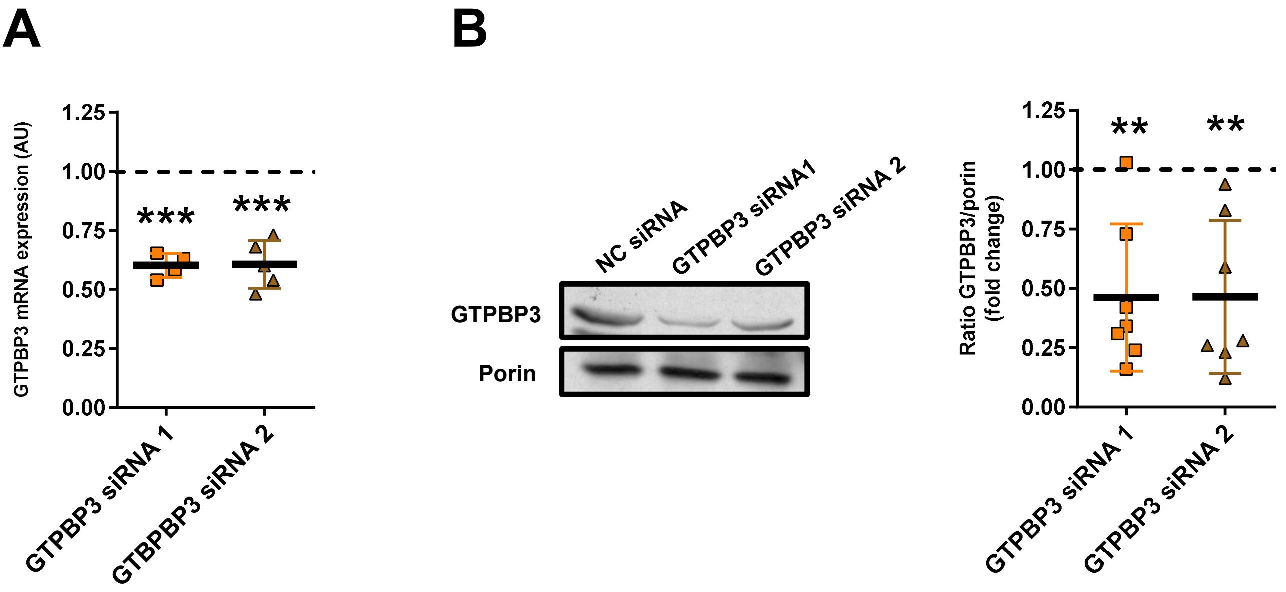

Figure S6. Transient silencing of GTPBP3 in osteosarcoma 143B cells. (A) qRT-PCR analysis of *GTPBP3* mRNA in GTPBP3 siRNA1- and GTPBP3 siRNA 2-transfected cells. **(B)** Representative immunoblots of GTPBP3 in GTPBP3 siRNA1-, GTPBP3 siRNA 2- and NC siRNA-transfected cells. Porin was used as a loading control. The scatter plot shows the densitometric analysis of GTPBP3 normalized to porin. Data represent the means ± SD from at least 3 independent determinations and are expressed as fold change respect to NC. Differences from NC values were found to be statistically significant at *$p<0.05$ and ***$p<0.001$.

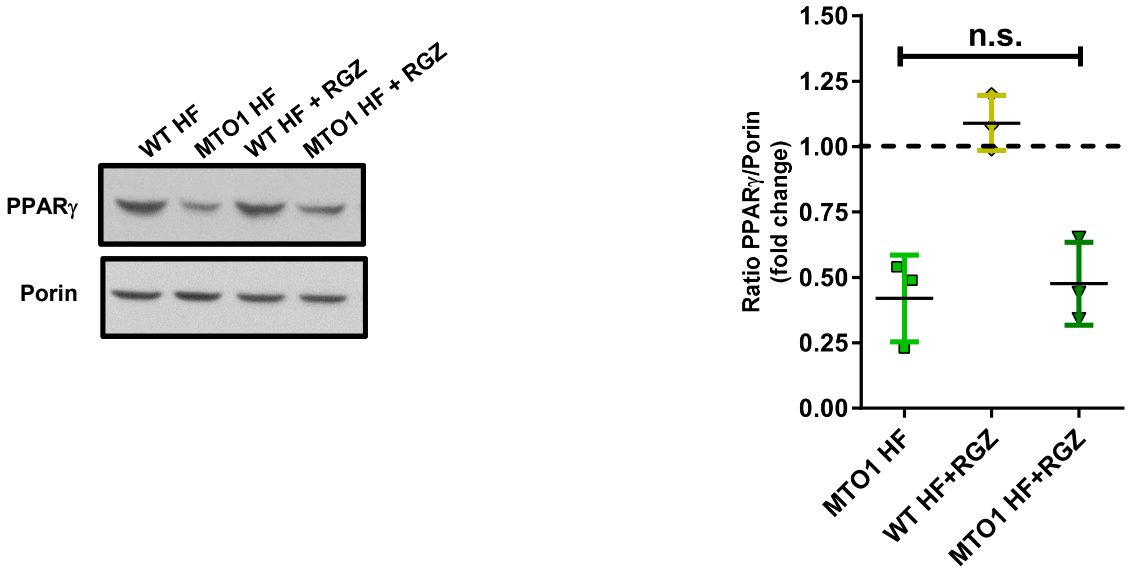

Figure S7. PPARγ expression is not affected by Rosiglitazone treatment. Representative immunoblot of PPARγ in WT and MTO1 HF, treated or not with 5 μM rosiglitazone (RGZ) for 1h. Porin was used as a loading control. The scatter plots show the densitometric analysis of PPARγ normalized to porin and represented as fold change relative to WT values. Data represent the means ± SD from 3 independent determinations and are expressed as fold change respect to WT HF values. n.s.: non-significant differences.

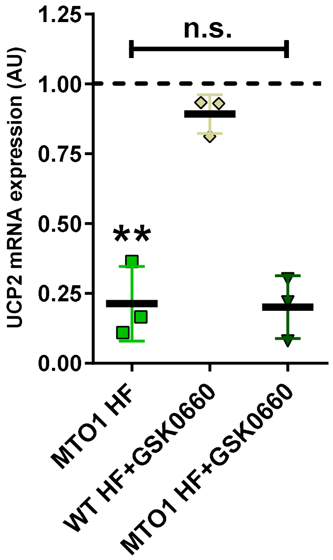

Figure S8. Reduction of the UCP2 expression in MTO1 HF does not depend on PPAR*ß/δ*. qRT-PCR of *UCP2* mRNA expression in MTO1 human fibroblasts (MTO1 HF), treated or not with 5 μM GSK0660, a PPARβ/δ antagonist, for 24h. Data represent the means ± SD from 3 independent determinations and are expressed as fold change respect to WT HF values. n.s.: non-significant differences.

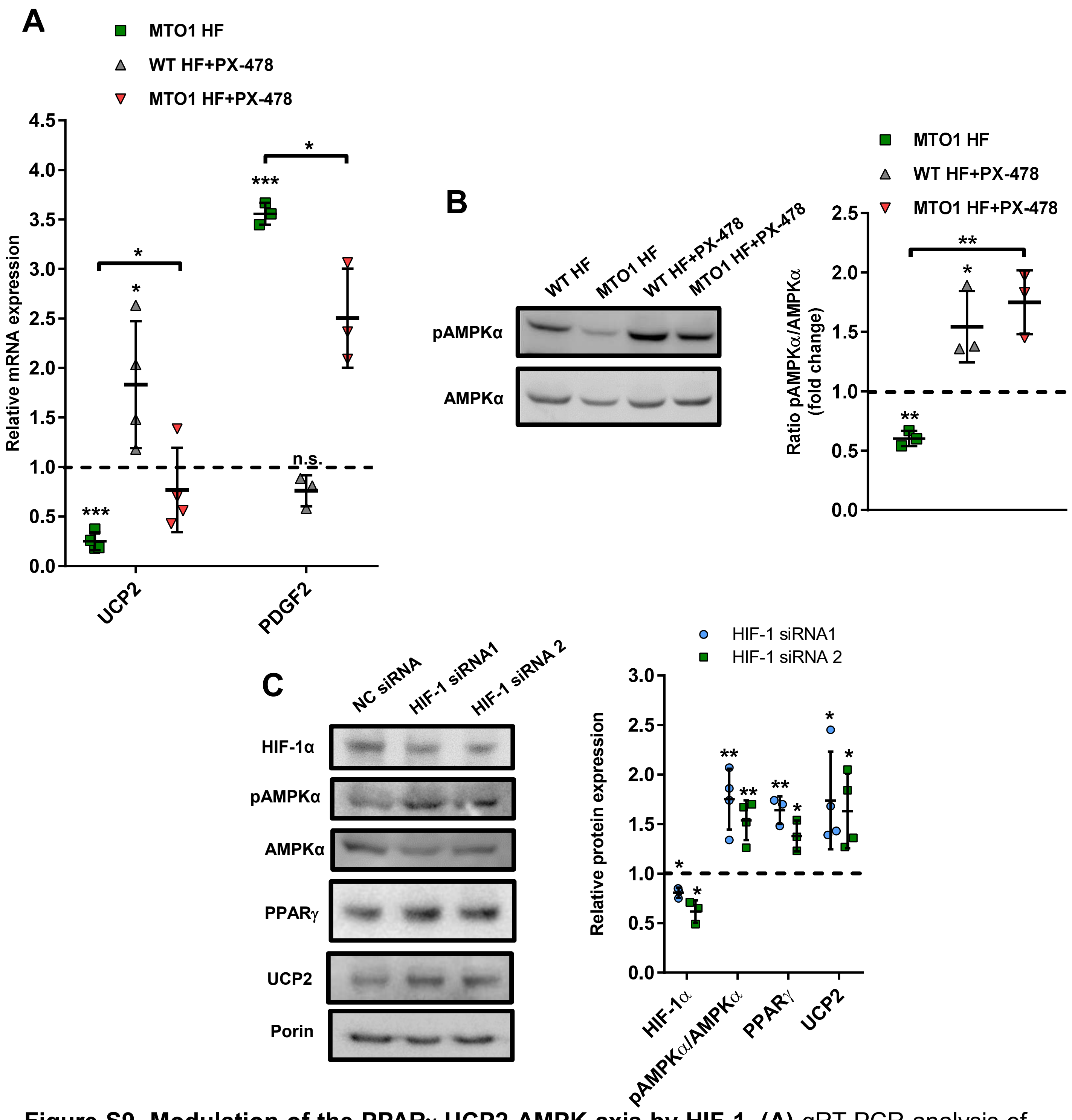

Figure S9. Modulation of the PPARγ-UCP2-AMPK axis by HIF-1. (A) qRT-PCR analysis of the *UCP2* and *PDGF2* mRNA expression in WT and MTO1 HF, treated or not with 25 μM PX-478 (HIF-1 inhibitor) for 24h. Data are represented as fold change respect to untreated WT HF values. **(B)** Representative immunoblots of phosphor-Thr172-AMPKα and AMPKα in WT and MTO1 HF, treated or not with 25 μM PX-478 for 24h. AMPKα was used as a loading control. The scatter plot shows densitometric analysis of phosphor-Thr172-AMPKα normalized to AMPKα, and represented as fold change relative to untreated WT HF values. **(C)** Representative immunoblots of HIF-1, phosphor-Thr172-AMPKα, AMPKα, PPARγ and UCP2 in HIF1 siRNA 1-, HIF1 siRNA 2- and NC siRNA-transfected 143B cells. Porin and AMPKα were used as loading controls. The scatter plot shows densitometric analysis of HIF-1, PPARγ and UCP2 normalized to porin and phosphor-Thr172-AMPKα normalized to AMPKα, and represented as fold change relative to NC. Data represent the means ± SD from at least 3 independent determinations and are expressed as fold change respect to NC. Differences from control values were found to be statistically significant at *p<0.05, **p<0.01 and ***p<0.001. n.s.: non-significant differences.

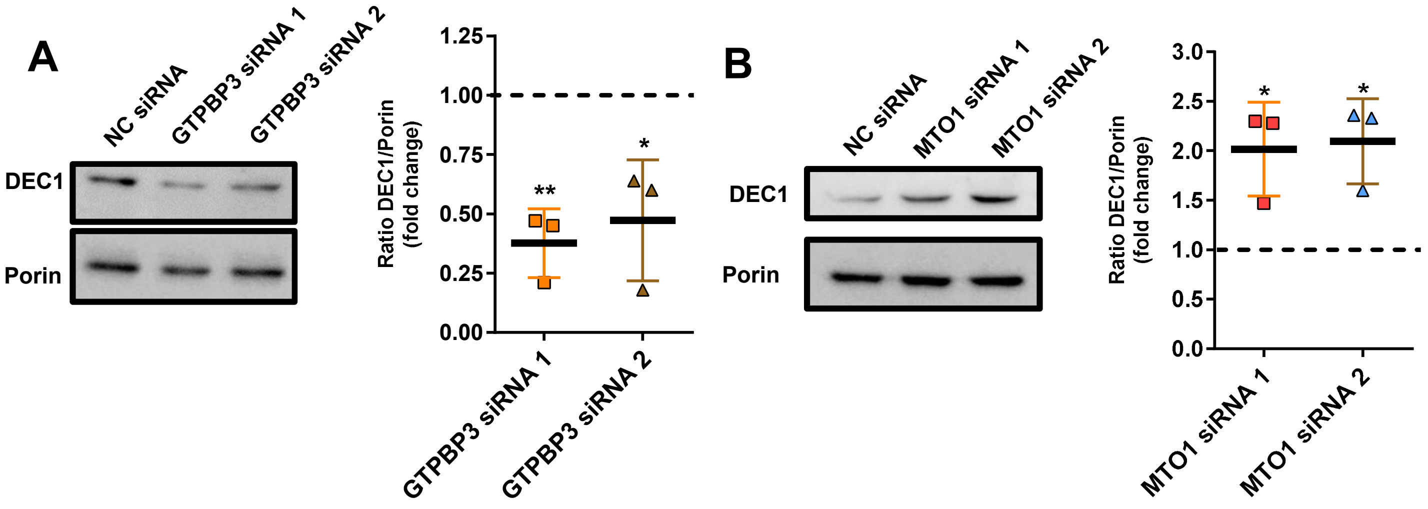

Figure S10. DEC1 expression in GTPBP3 and MTO1 knock-down cells. (A and B) Representative immunoblots of DEC1 in GTPBP3 siRNA 1-, GTPBP3 siRNA 2- and NC siRNA-transfected 143B cells (A), and in MTO1 siRNA 1-, MTO1 siRNA 2- and NC siRNA-transfected 143B cells (B). Porin was used as loading control. The scatter plot shows densitometric analysis of DEC1 normalized to porin, and represented as fold change relative to NC values. All data are the mean ± SD of at least three different experiments. Differences from control values were found to be statistically significant at *p<0.05 and **p<0.01.

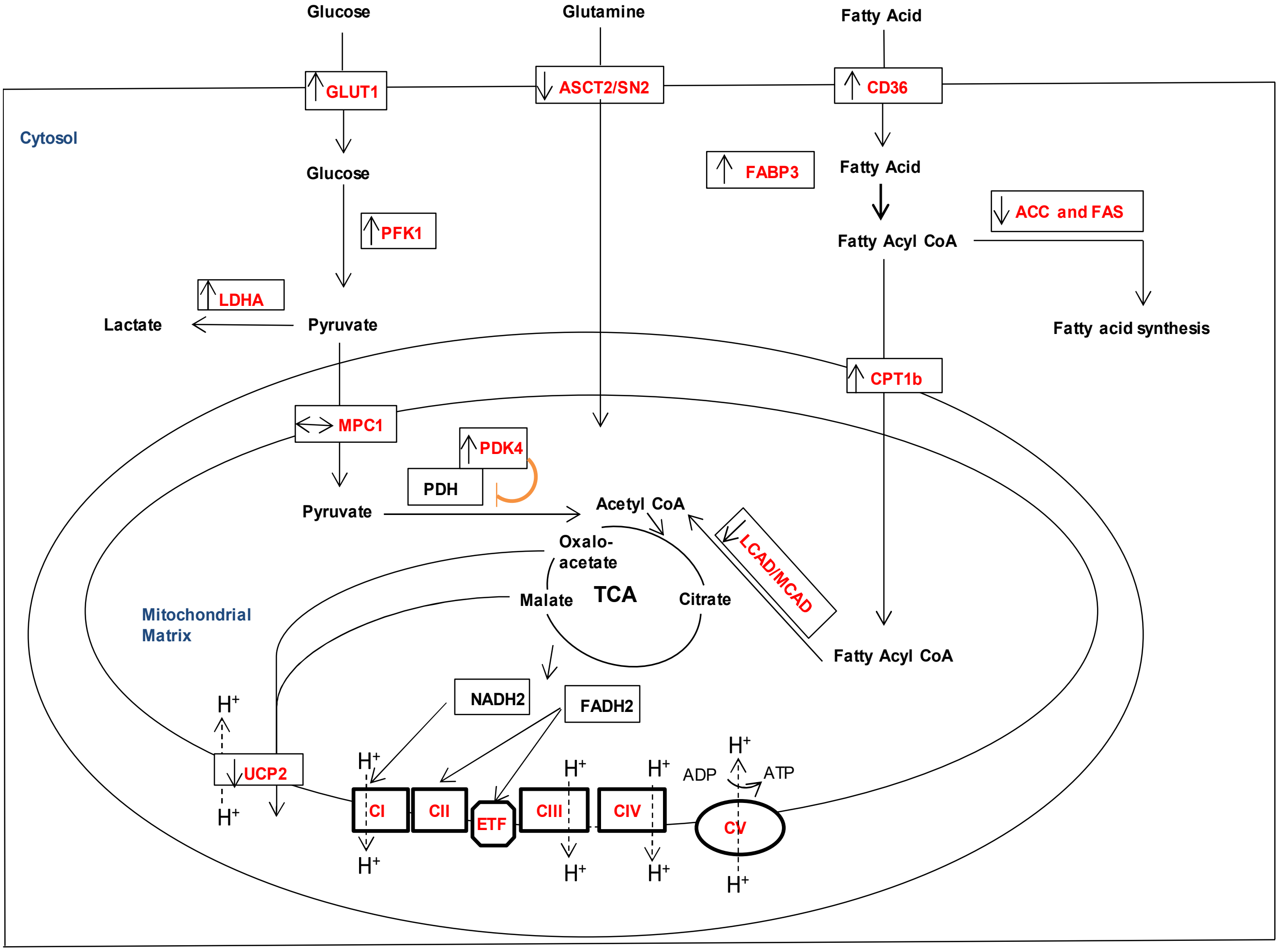

Figure S11. Scheme of metabolic pathways affected by the MTO1 defect. The expression of selected genes in MTO1 fibroblasts suggests severe metabolism changes, which would be represented by increased glycolysis, reduced pyruvate oxidation, increased lipid uptake, decreased *de novo* FA synthesis, and decreased FA oxidation. Arrows within boxes represent up- or down-regulation of the indicated gene.

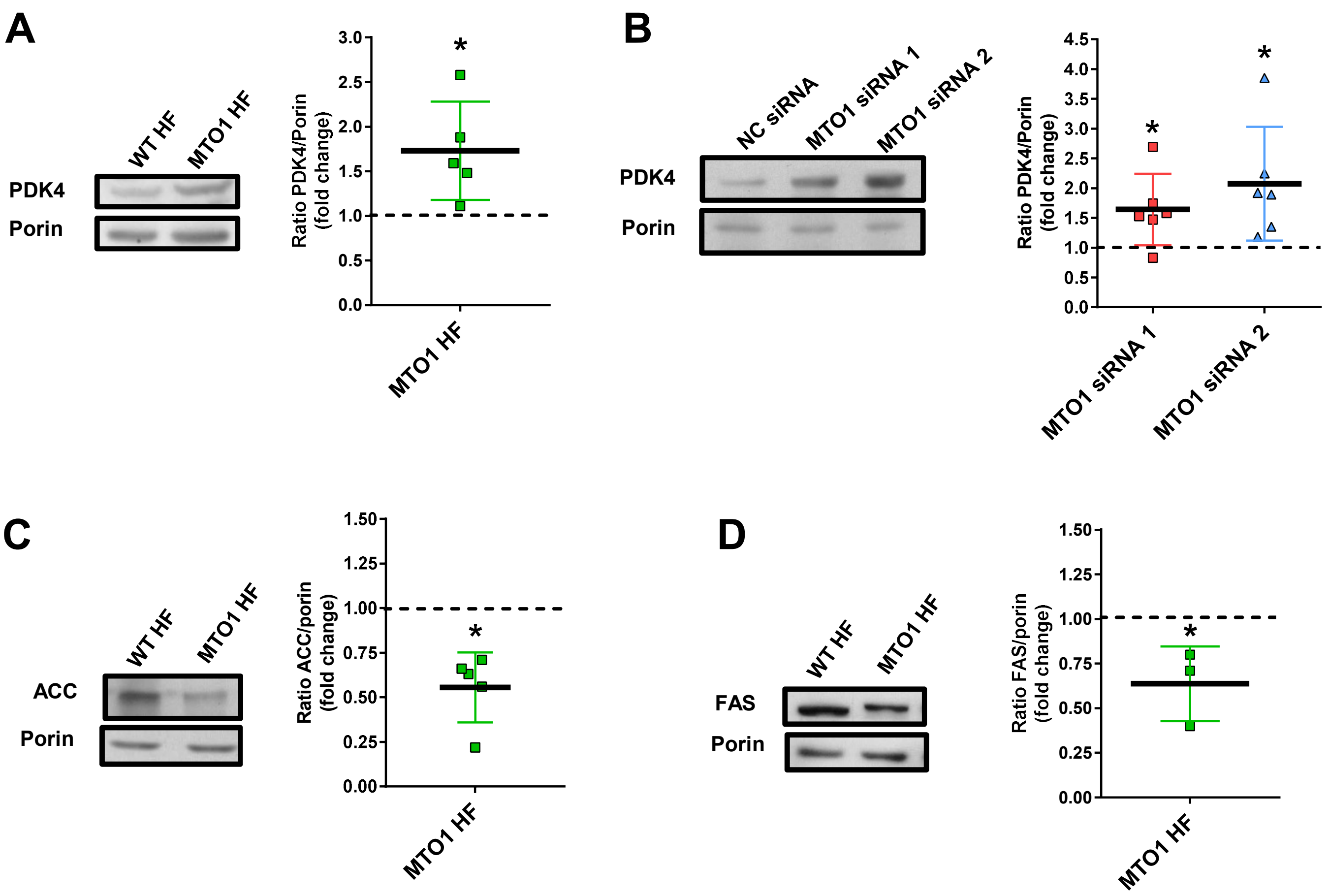

Figure S12. Expression profile of metabolism genes in MTO1-defective cells. (A and B) Representative immunoblots of PDK4 in WT and MTO1 HF (**A**), and in MTO1-silenced 143B cells **(B)**. Porin was used as a loading control. The scatter plots show the densitometric analysis of PDK4 normalized to porin and represented as fold change relative to WT or NC values. **(C and D)** Representative immunoblots of ACC **(C)** and FAS **(D)** in WT and MTO1 HF. The membranes were also probed with an antibody against porin, which was used as a loading control. The scatter plots show the densitometric analysis of ACC and FAS normalized to porin and represented as fold change relative to WT. All data are the mean ± SD of at least three different experiments. Differences from WT or NC values were found to be statistically significant at *p<0.05 and ***p<0.001. NC siRNA: negative control siRNA.

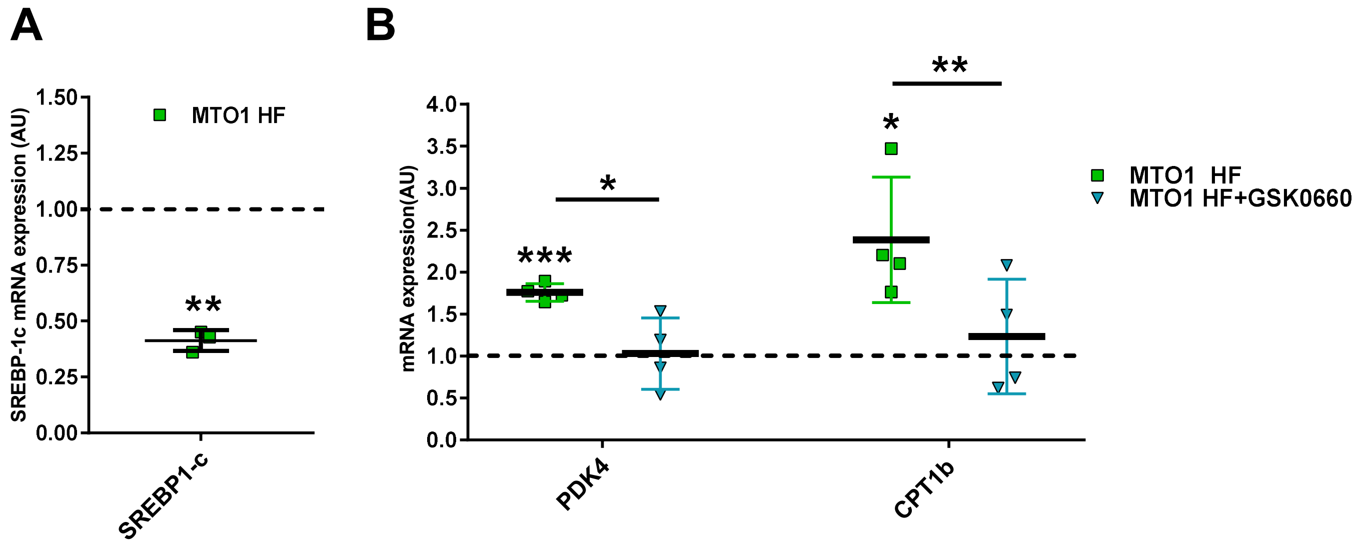

Figure S13. Expression of SREBP-1c, PDK4 and CPT1b in MTO1 fibroblasts. (A) qRT-PCR analysis of *SREBP-1c* mRNA expression in MTO1 human fibroblasts (MTO1 HF). **(B)** qRT-PCR analysis of *PDK4* and *CPT1b* mRNA expression in MTO1 HF treated or not with 5 µM GSK0660 (PPARß antagonist) for 24 h. Data represent the means ± SD from at least 3 independent determinations and are expressed as fold change respect to WT HF. Differences from WT values were found to be statistically significant at *p<0.05,**p>0.01 and ***p<0.001.

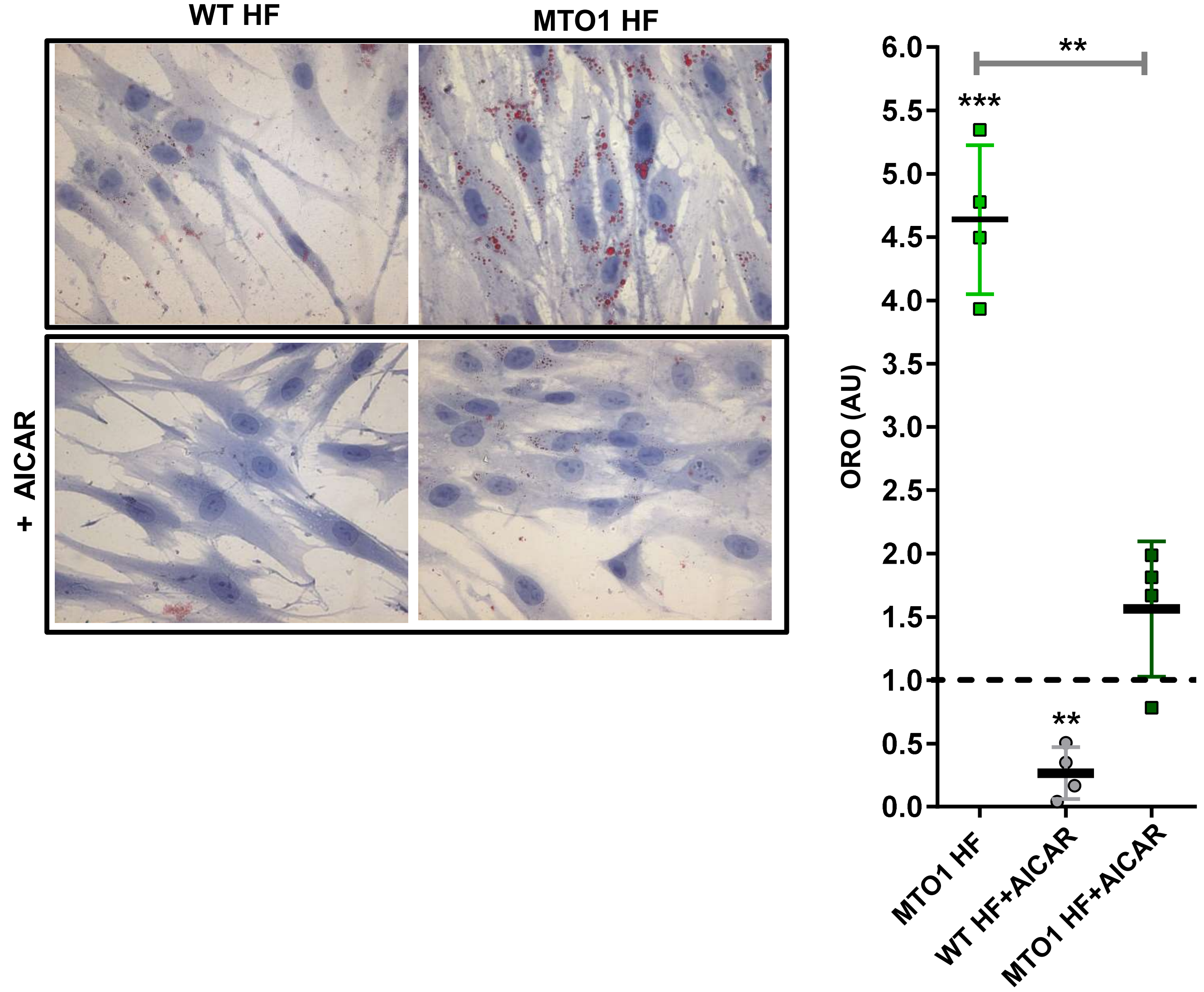

Figure S14. AICAR treatment reduces lipid accumulation in MTO1 fibroblasts. (A) Representative microscope pics of intracellular lipid droplets stained with Oil Red O (ORO) in wild-type (WT HF) and MTO1 (MTO1 HF) human fibroblasts under normal conditions (top panels) and treated with 1mM AICAR for 1h (bottom panels). **(B)** Ratio of cells with lipid droplets (red) in relation to the total number of cells. Results are expressed as fold change relative to WT HF. Data represent the means ± SD from at least 3 independent determinations. Differences from WT HF values were found to be statistically significant at **p<0.01 and ***p<0.001.

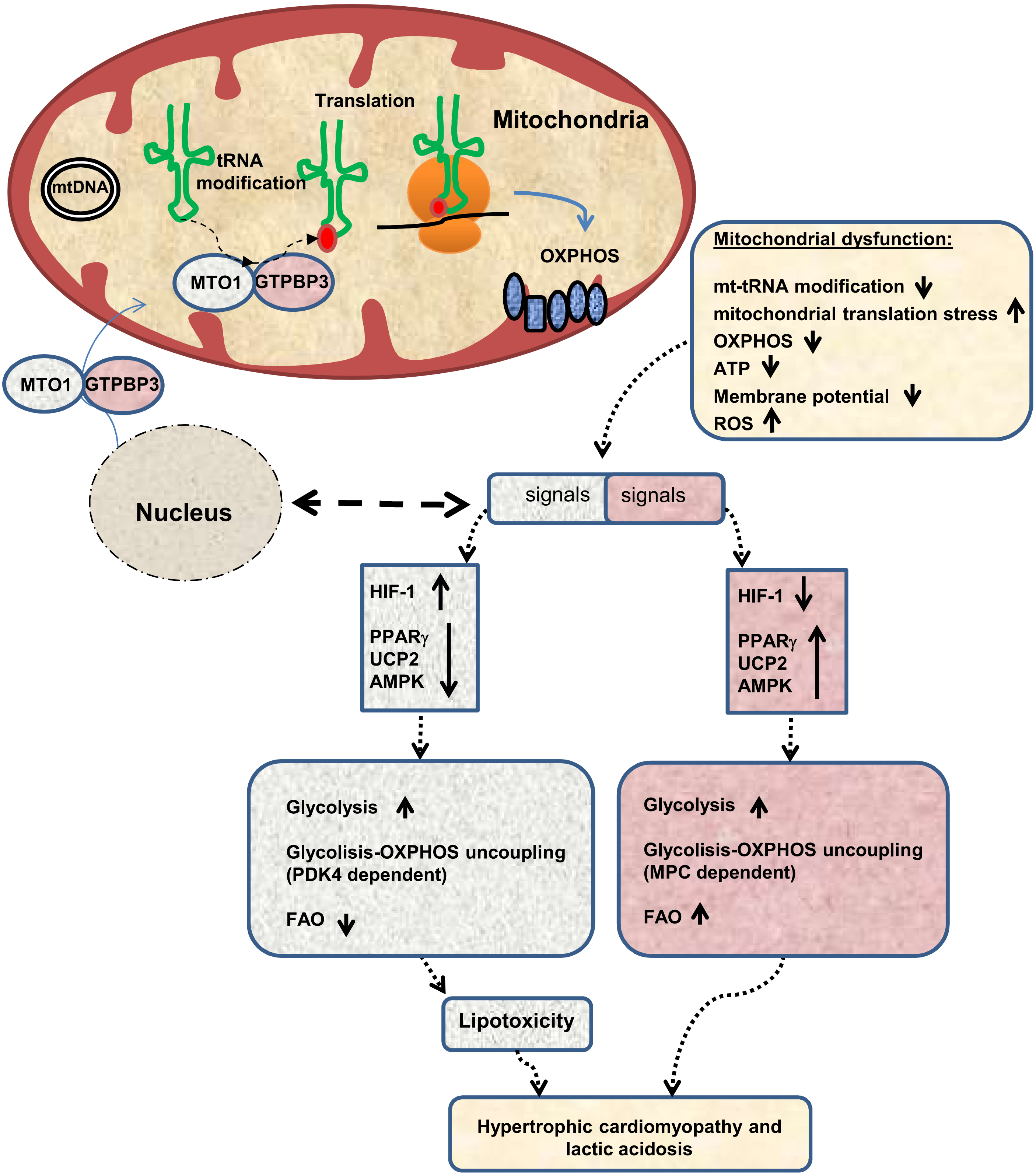

Figure S15. Proposed pathological mechanisms in MTO1 and GTPBP3 defective cells. MTO1 and GTPBP3 are jointly responsible for the synthesis of the taurinomethyl group at position 5 of U34 (τm^5U) in mt-tRNAs. However, one of them (likely MTO1) could have an additional function (ribosome biogenesis?). Defects in MTO1 or GTPBP3 affect differently mitochondrial translation and/or OXPHOS function, leading to opposite effects on the HIF-PPARγ-UCP2-AMPK axis and, accordingly, on fatty acid metabolism. Lipotoxicity may be an underlying factor in the development of the hypertrophic cardiomyopathy in MTO1 patients.

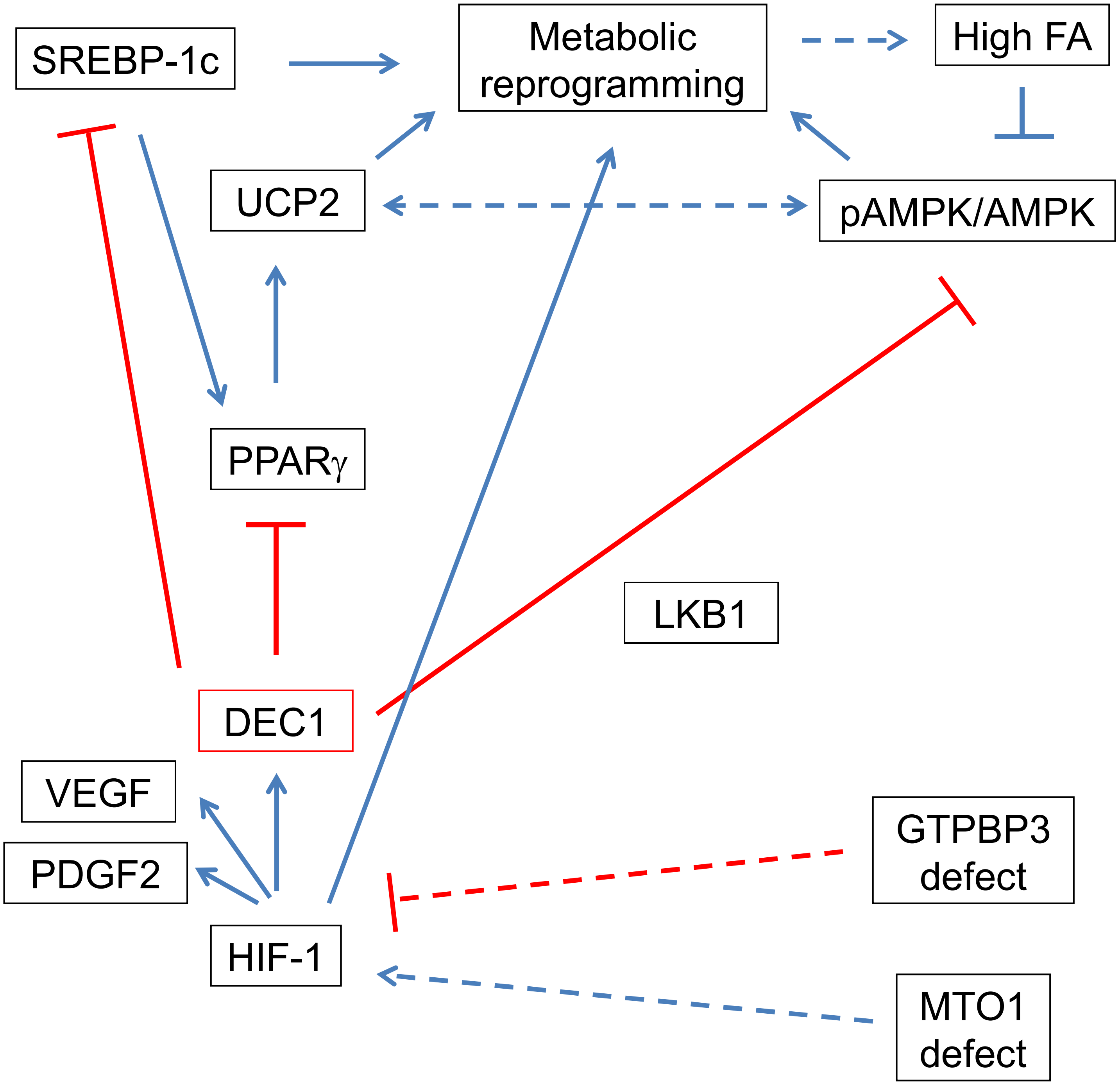

Figure S16. A working model of the signaling pathways operating in MTO1- and GTPBP3-defective cells. Dashed lines indicate that the link may be no direct.

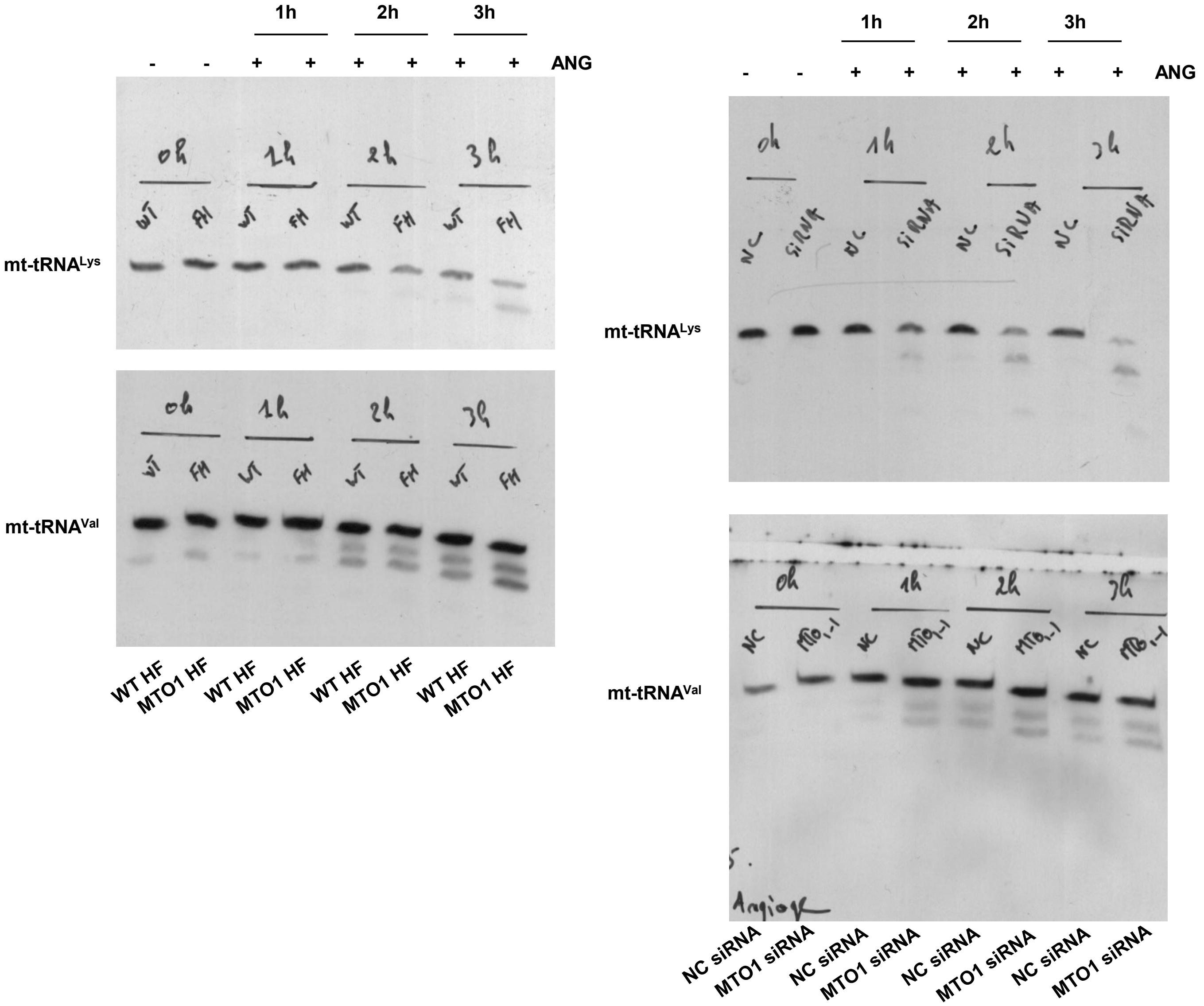

Figure S17. Full-length Northern blots of mt-tRNA^{lys} and mt-tRNA^{Val} molecules after in vitro angiogenin (ANG) digestion of small RNAs purified from WT HF and MTO1 HF (shown in Fig. 1A), and from MTO1 siRNA 1- and Negative Control (NC) siRNA-transfected cells (shown in Fig. 1B) for 1, 2 and 3 h.

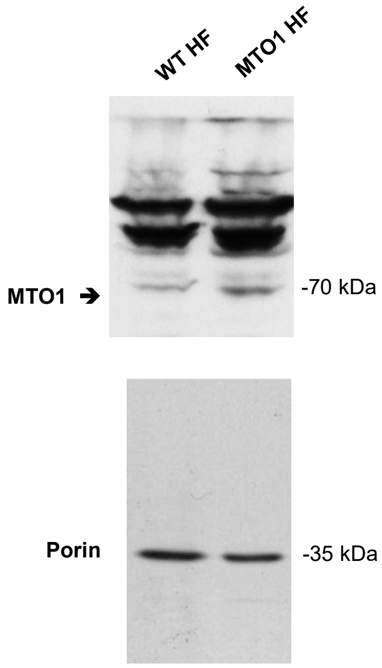

Figure S18. Full-length Western blots of MTO1 and Porin in wild-type (WT HF) and MTO1 (MTO1 HF) human fibroblasts (shown in Fig. 1D). The recognition of the MTO1 band by the anti-MTO1 antibody has been previously demostrated by MTO1 silencing[21]. The full-length membrane was cut and incubated with the respective antibody.

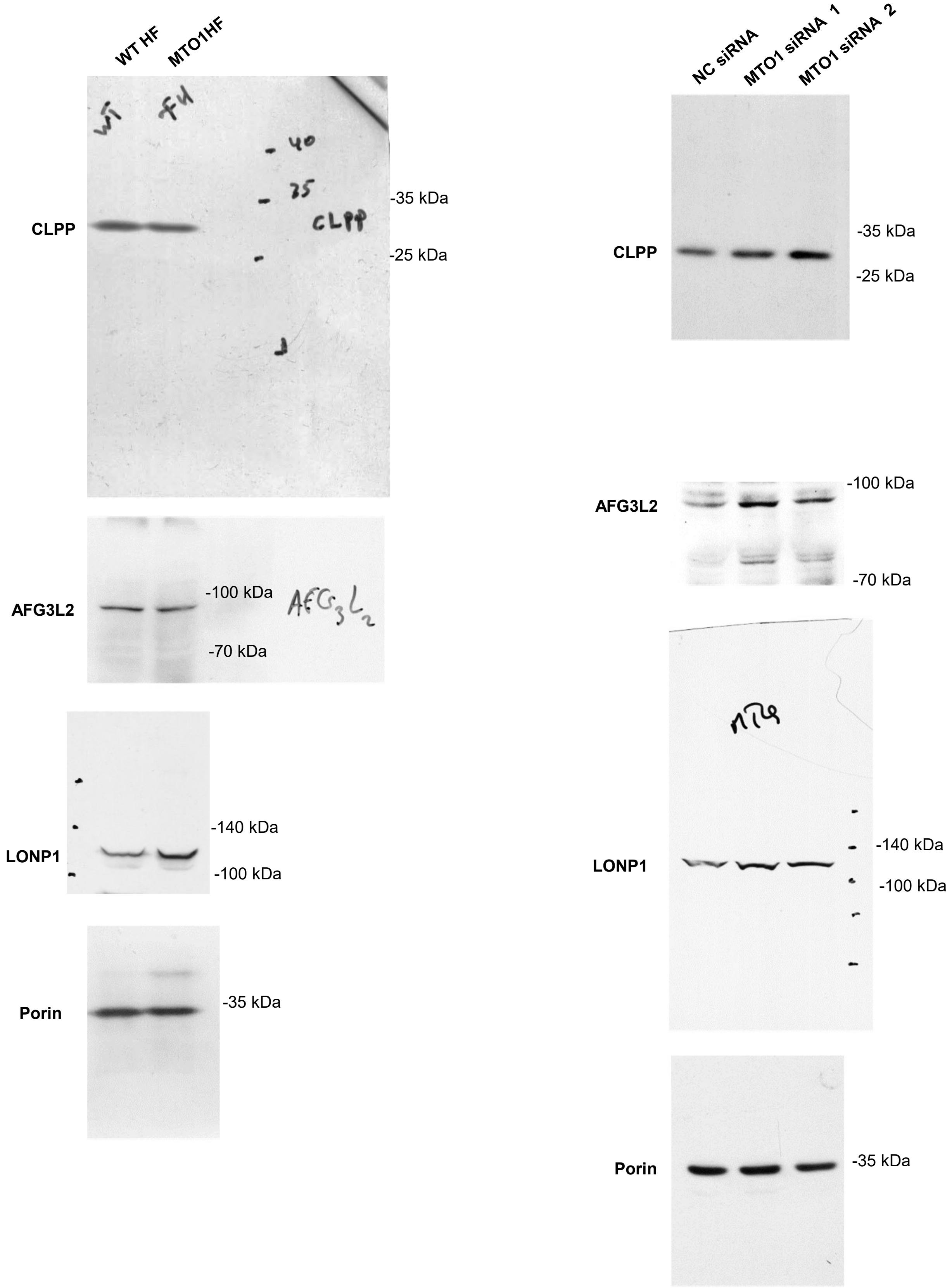

Figure S19. Full-length Western blots of Figures 2A and 2B. The full-length membrane was cut and incubated with the indicated antibody.

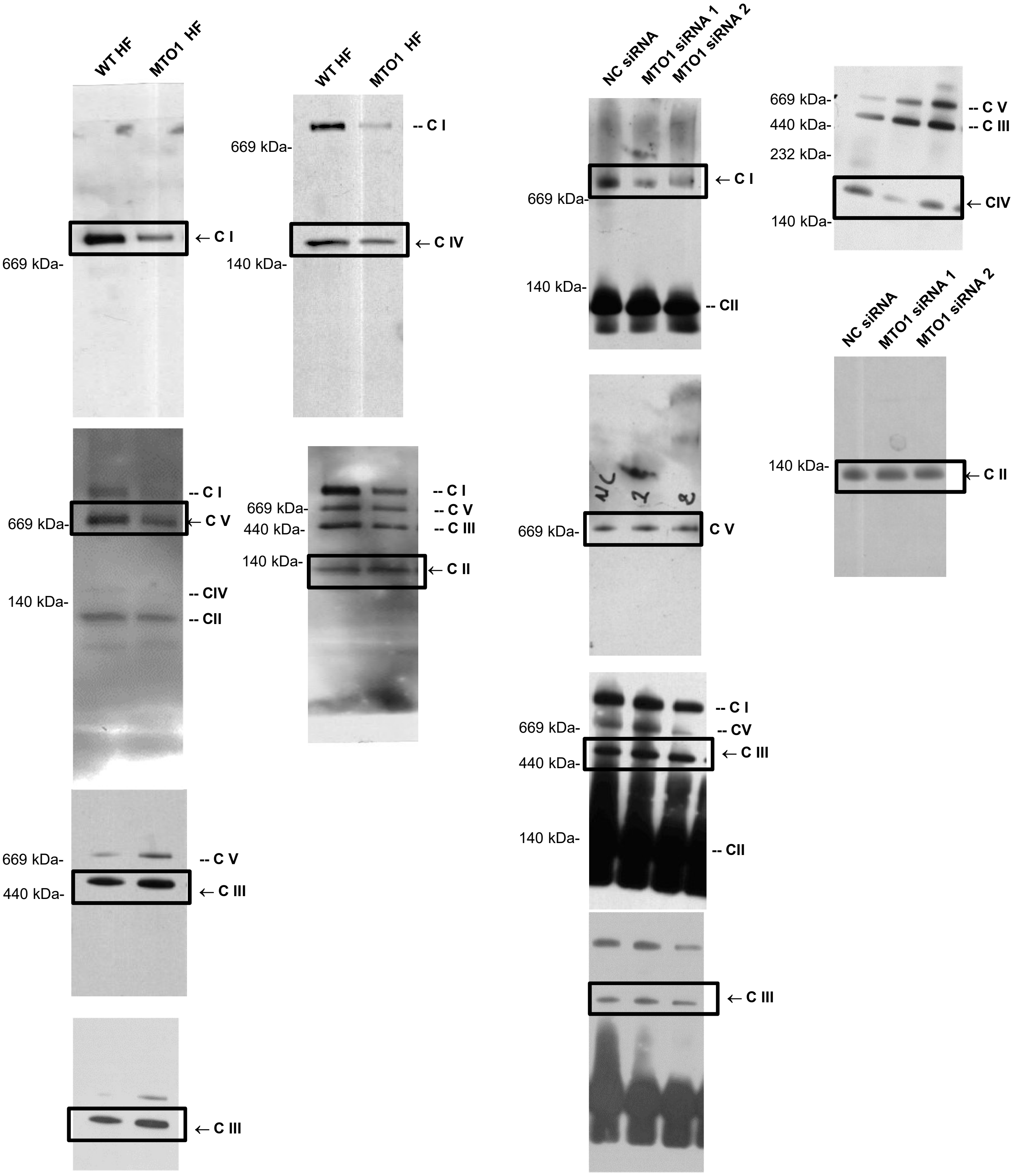

Figure S20. Full-length Western blots of Figures 2B and 2C. Representative blots from independent experiments are shown. The presence of additional bands besides the expected one (highlighted with a rectangle) after the incubation with a specific antibody is due to previous antibody incubations. A blot of CIII with less exposure time is shown at the bottom.

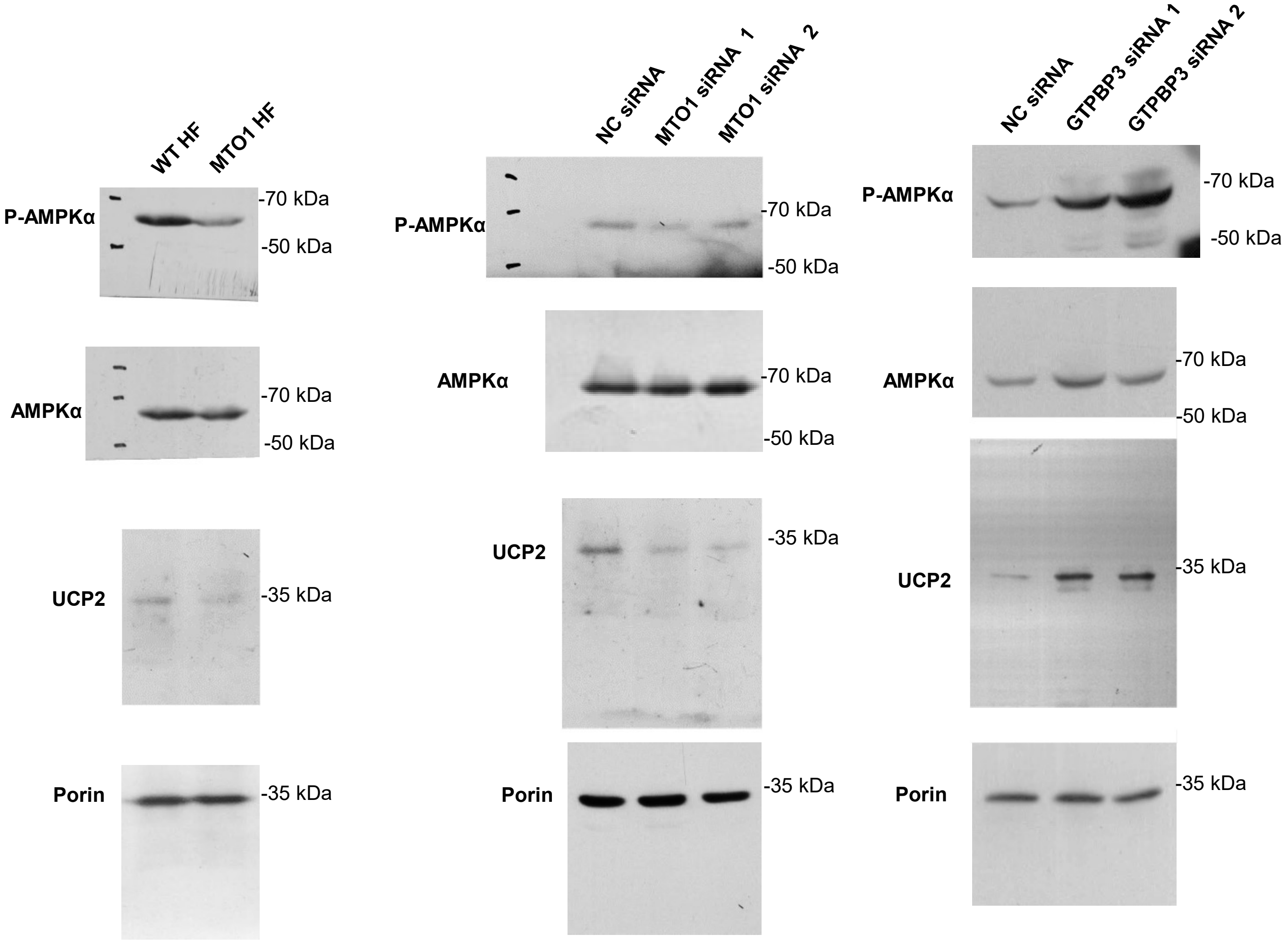

Figure S21. Full-length western blots of phospho-Thr172-AMPKα, AMPKα, UCP2 and Porin in wild-type (WT HF) and MTO1 (MTO1 HF) human fibroblasts (shown in Fig. 3A), in MTO1 siRNA1-, MTO1 siRNA 2- and NC siRNA-transfected cells (shown in Fig. 3E), and in GTPBP3 siRNA1-, GTPBP3 siRNA 2- and NC siRNA-transfected cells (shown in Fig. 3I). The full-length membrane was cut and incubated with the indicated antibody.

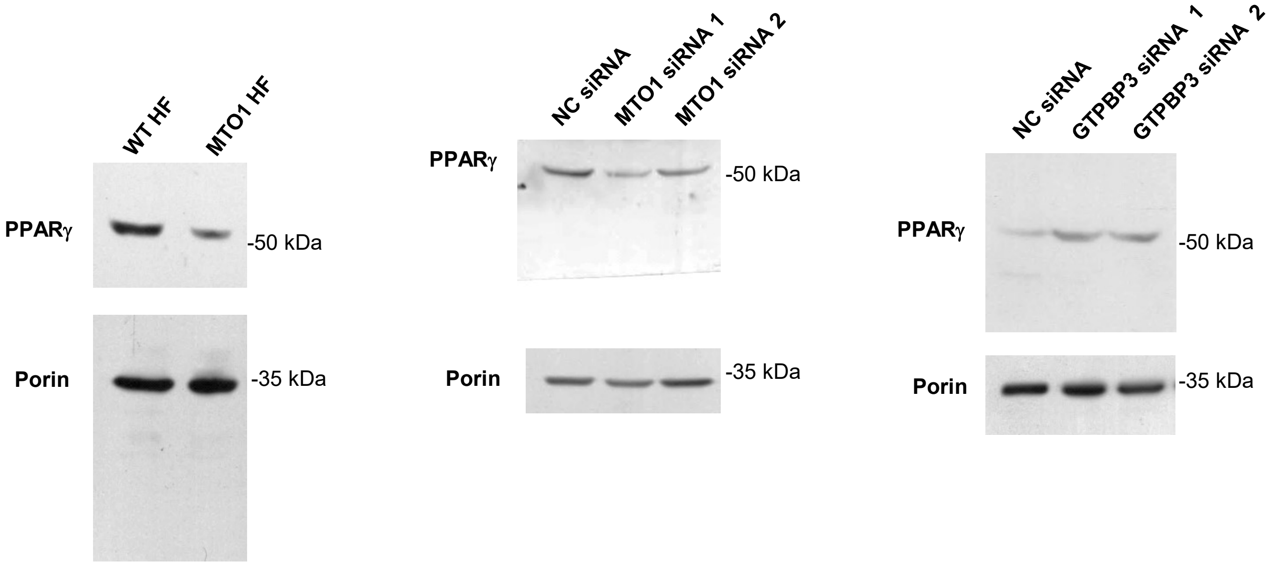

Figure S22. Full-length Western blots of PPARγ in WT and MTO1 HF (shown in Fig.4B), in MTO1 siRNA1-, MTO1 siRNA 2- and NC siRNA-transfected cells (shown in Fig. 4C), and in GTPBP3 siRNA1-, GTPBP3 siRNA 2- and NC siRNA-transfected cells (shown in Fig. 4D). The full-length membrane was cut and incubated with the respective antibody.

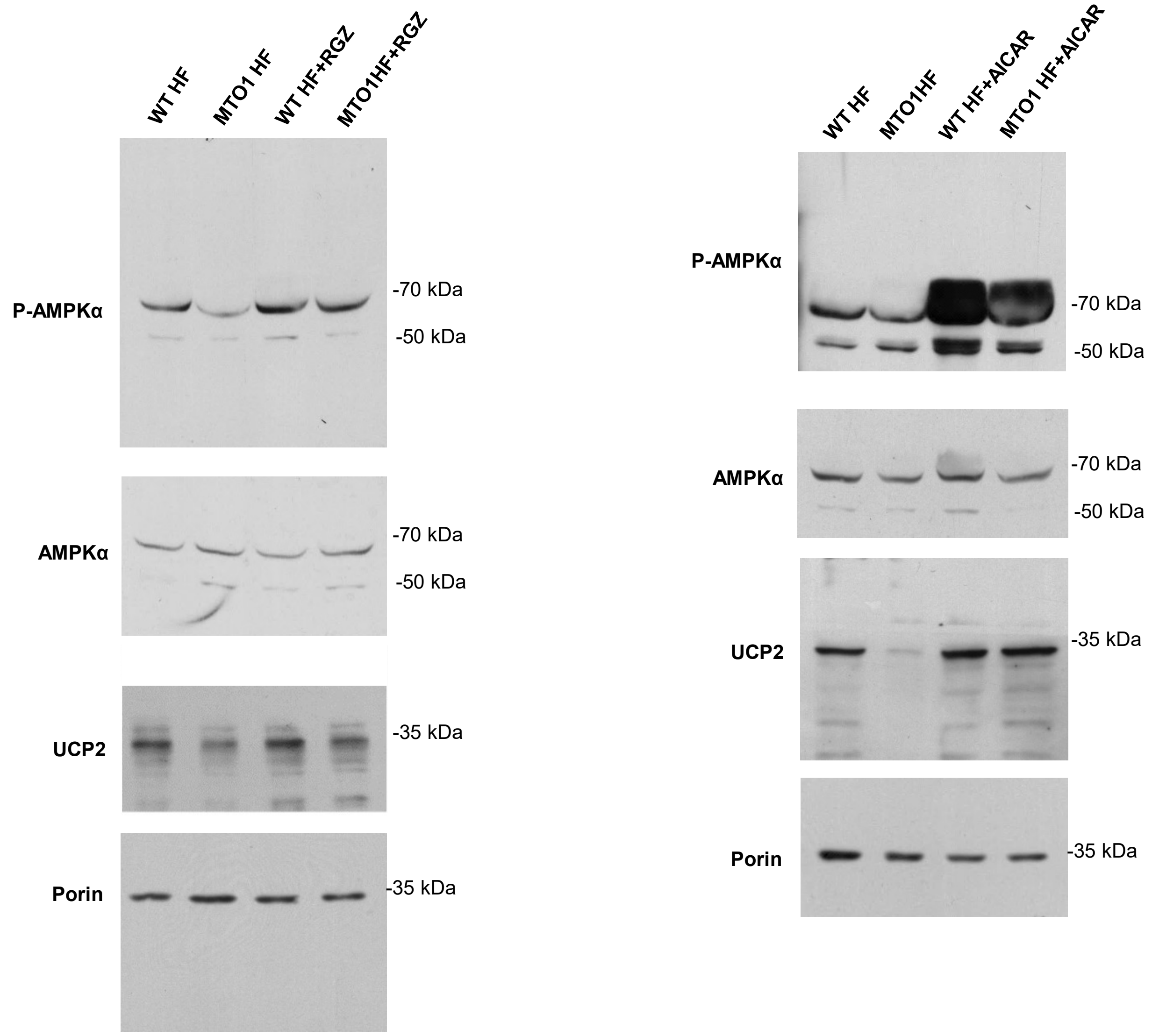

Figure S23. Full-length Western blots of phosphor-Thr172-AMPKα, AMPKα, UCP2 and Porin in WT and MTO1 HF, treated or not with 5 μM RGZ (shown in Fig. 5B) or 1 mM AICAR for 1h (shown in Fig. 5C). The full-length membrane was cut and incubated with the indicated antibody.

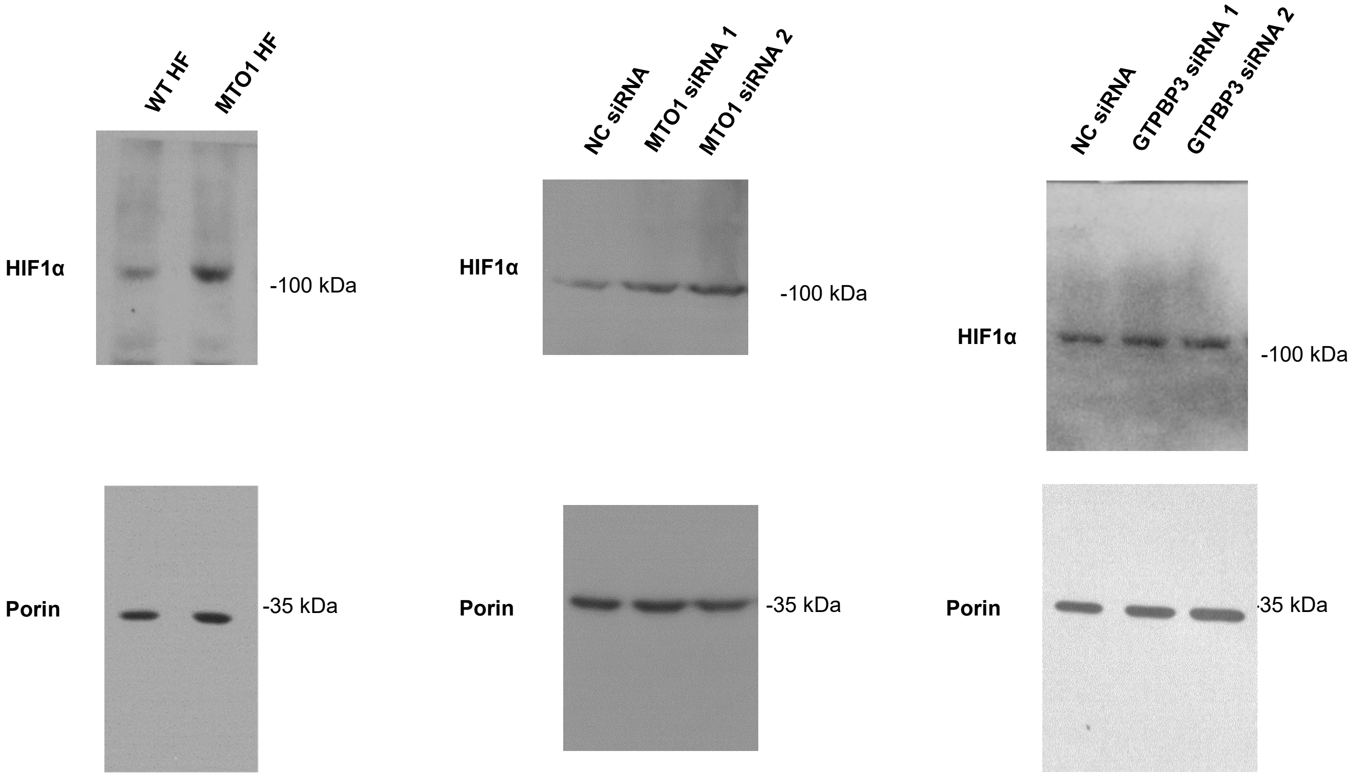

Figure S24. Full-length Western blots of HIF1α and DEC1 in WT, MTO1 HF (A), in MTO1 siRNA 1-, MTO1 siRNA-2 and NC siRNA-transfected cells (B), and in GTPBP3 siRNA-1, GTPBP3 siRNA-2 and NC siRNA-transfected cells (shown in Fig. 6). The full-length membrane was cut and incubated with the indicated antibody.

Table S1. In vivo complementation analysis of the wild-type and mutant MnmG proteins. HPLC analysis of the nucleoside composition from bulk tRNA purified from the *E.coli* strains MG1655 (wild-type) and IC5241 (*mnmG::Tn10*), which was transformed with the empty plasmid (pBAD22), or pIC1180 (pBAD22 expressing the MnmG wild-type protein), or pIC1750 (pBAD22 expressing MnmG-Arg427Cys). [a]Levels of nucleoside s^2U detected in the HPLC analysis. –ARA and +ARA indicate, respectively, the absence or presence of the inducer arabinose in the growth medium. s^2 is the modification that remains in U34 (at position 2) in the absence of the MnmG-dependent modification (at position 5). s^4 is a nucleoside independent of the MnmG activity and used herein as a reference. The numbers are calculated as the absorbance of s^2U relative to the absorbance of s^4U at 314 nm and are the mean from at least two independent experiments. The asterisks indicate that s^2U was undetectable since U34 was fully modified at both position 2 and 5 (predominant nucleoside: mnm^5s^2U).

Strain/plasmid	MnmG protein	s^2U/s^4U ratio[a]	
		- ARA	+ARA
**MG1655**	Wild type	0.000*	0.000*
**IC5241/pBAD22**	None	0.023	0.030
**IC5241/pIC1180**	Flag-MnmG	0.007	0.000*
**IC5241/pIC1750**	Arg464Cys	0.025	0.030

Table S2. Enzymatic activity of Respiratory Chain Complexes in the muscle biopsy of the MTO1 patient. *Activity of citrate synthase (CS) and the specified complex normalized to total citrate synthase activity (U/cU CS).

	Activity*	Decision level*
Citrate synthase (CS)	2372.7 U/L	900
Complex I/CS	……	10
Complex II/CS	9.7	4.5
Complex III/CS	18.1	31
Complex IV/CS	12.7	30
Complex I+III/CS	5.2	16
Complex II+III/CS	11.9	4.5

Table S3. List of the oligonucleotide sequences used in this work.

Fw and Rv denote forward and reverse primer, respectively.

Gene name	Oligonucleotides name	Sequence (5'→3')	Assay
**GTPBP3**	GTPBP3-Sab	GTPBP3 SaBioscience RT^{2}qPCR primer assay	qRT-PCR
**MTO1**	MTO1-Fw	CCTGAAGGAATGGATTCTGAC	qRT-PCR
	MTO1-Rv	GCTGCAGCTTCCTCATAACC	qRT-PCR
**ACTB**	ACTB-Fw	TGAGCGCGGCTACAGCTT	qRT-PCR
	ACTB-Rv	TCCTTAATGTCACGCACGATT	qRT-PCR
**Thioxiredoxin-1**	Thioxiredoxin-1-Fw	GCCTTGCAAAATGATCAAGC	qRT-PCR
	Thioxiredoxin-1-Rv	TTGGCTCCAGAAAATTCACC	qRT-PCR
**Thioxiredoxin-2**	Thioxiredoxin-2-Fw	CGCCATTGAGTATGAGGTGTCA	qRT-PCR
	Thioxiredoxin-2-Rv	CCACCACGTCCCCATTCTT	qRT-PCR
**Peroxiredoxin-3**	Peroxiredoxin-3-Fw	GGCGTTCCAGTATGTAGAAACACA	qRT-PCR
	Peroxiredoxin-3-Rv	GCTGGACTTGGCTTGATCGT	qRT-PCR
**Peroxiredoxin-5**	Peroxiredoxin-5-Fw	CAAGGCGGAAGGCAAGGT	qRT-PCR
	Peroxiredoxin-5-Rv	CACCAGCGAATCATCTAGTAATAAGTCT	qRT-PCR
**UCP2**	UCP2-Fw	CATCGGCCTGTATGATTCTG	qRT-PCR
	UCP2-Rv	TGGAATCGGACCTTTACCAC	qRT-PCR
**PPAR-α**	PPARα-Fw	CTTCAACATGAACAAGGTCAAAGC	qRT-PCR
	PPARα-Rv	AGCCATACACAGTGTCTCCATATCA	qRT-PCR
**PPAR-ß**	PPARß-Fw	TCAGAAGAAGAACCGCAAC	qRT-PCR
	PPARß-Rv	TAGGCATTGTAGATGTGCTTGG	qRT-PCR
**PPAR-γ**	PPARY-Fw	GAAACTTCAAGAGTACCAAAGTGCAA	qRT-PCR
	PPARY-Rv	AGGCTTATTGTAGAGCTGAGTCTTCTC	qRT-PCR
**GLUT1**	GLUT1-Fw	CATCAACGCTGTCTTCTATTACTC	qRT-PCR
	GLUT1-Rv	ATGCTCAGATAGGACATCCA	qRT-PCR
**PFK1**	PFK1-Fw	GCCGACTGGGTTTTTATTCCT	qRT-PCR
	PFK1-Rv	ACGAGAACCACGGGTCCTT	qRT-PCR
**LDHA**	LDHA-Fw	GCCTGTATGGAGTGGAATGAA	qRT-PCR
	LDHA-Rv	CCAGGATGTGTAGCCTTTGAG	qRT-PCR
**LDHB**	LDHB-Fw	GGGAAAGTCTCTGGCTGATGAA	qRT-PCR
	LDHB-Rv	CTGTCACAGAGTAATCTTTATCGGC	qRT-PCR
**PDK4**	PDK4-Fw	GGGTCTCAATAGTGTCACC	qRT-PCR
	PDK4-Rv	GTGGGCCTGGGCATTTAGCA	qRT-PCR
**MPC1**	MPC1-Fw	TGACATTCATGAGATTTGCCTACA	qRT-PCR
	MPC1-Rv	TGAGCTGGGCTACTTCATTTGTT	qRT-PCR
**CD36**	CD36-Fw	TGCCTCTCCAGTTGAAAACC	qRT-PCR
	CD36-Rv	CACAGGTCTCCCTTCTTTGC	qRT-PCR
**FABP3**	FABP3-Fw	ACTTGTGCGGGAGCTAATTG	qRT-PCR
	FABP3-Rv	TAAGTGCGAGTGCAAACTGC	qRT-PCR
**CPT1a**	CPT1a-Fw	TCCTTCCAACTCACATTCAG	qRT-PCR
	CPT1a-Rv	GGTGTCTGTCTCCTCTCC	qRT-PCR
**CPT1b**	CPT1b-Fw	GGTCGACTTCCAGCTCAGTC	qRT-PCR
	CPT1b-Rv	CAGGAGGAACCCACTGTTGT	qRT-PCR
**LCAD**	LCAD-Fw	AAGTGATGTTGTGATTGTAGTTG	qRT-PCR

	LCAD-Rv	GAATAGTTCTGCGGTATCCTG	qRT-PCR
**MCAD**	MCAD-Fw	AATTAGTGAAGAATTGGCTTATGG	qRT-PCR
	MCAD-Rv	ACATCAATGGCTCCTCAGTC	qRT-PCR
**HADH**	HADH-Fw	AGGGGAAGGTCATCATTGTG	qRT-PCR
	HADH-Rv	TGGAGGATTCGGATGACTTC	qRT-PCR
**ACC**	ACC-FW	GCCTCTTCCTGACAAACGAG	qRT-PCR
	ACC-Rv	TCCATACGCCTGAAACATGA	qRT-PCR
**FAS**	FAS-Fw	TATGCTTCTTCGTGCAGCAGTT	qRT-PCR
	FAS-Rv	GCTGCCACACGCTCCTCTAG	qRT-PCR
**ASCT2**	ASCT2-Fw	GAGGAATATCACCGGAACCA	qRT-PCR
	ASCT2-Rv	AGGATGTTCATCCCCTCCA	qRT-PCR
**SN2**	SN2-Fw	GAGTTGCGGCCACTTCAG	qRT-PCR
	SN2-Rv	TCCATTCATCTTTGGATCCTG	qRT-PCR
**GLS**	GLS-Fw	GCATACACTGGAGATGTGTCTGC	qRT-PCR
	GLS-Rv	TGTCCAAAGTGTAGTGCTTCATCC	qRT-PCR
**VEGF**	VEGF-Fw	TTGCTGCTCTACCTCCAC	qRT-PCR
	VEGF-Rv	GATGTCCACCAGGGTCTC	qRT-PCR
**PDGF2**	PDGF2-Fw	TCTGCTGCTACCTGCGTCTG	qRT-PCR
	PDGF2-Rv	AGAGTGGGAGCGGGTCAT	qRT-PCR
**SREBP-1c**	SREBP-1C-Fw	ACAGCCCACAACGCCATT	qRT-PCR
	SREBP-1C-Rv	TGCGCAAGACAGCAGATTT	qRT-PCR
**HIF-1α**	HIF-1α-Fw	AAGGTATTGCACTGCACAGG	qRT-PCR
	HIF-1α-Rv	AAATCAGCACCAAGCAGGTC	qRT-PCR
**DEC1**	DEC1-Fw	CCTTGAAGCATGTGAAAGCA	qRT-PCR
	DEC1-Rv	GCTTGGCCAGATACTGAAGC	qRT-PCR
**COX II**	COX II-Fw	CGATCCCTCCCTTACCATCA	mtDNA copy number quantification
	COXII-Rv	CCGTAGTCGGTGTACTCGTAGGT	mtDNA copy number quantification
**SDHA**	SDHA-Fw	TCTCCAGTGGCCAACAGTGTT	mtDNA copy number quantification
	SDHA-Rv	GCCCTCTTGTTCCCATCAAC	mtDNA copy number quantification
**mt-tRNA^{Lys}**	mt-tRNA^{Lys} dig	TGGTCACTGTAAAGAGGTGTTGGT	Northern blot analysis
**mt-tRNA^{Val}**	mt-tRNA^{Val} dig	GAAATCTCCTAAGTGTAAGTTGGGTGCTTTG	Northern blot analysis

Supplementary References

1 Semenza, G. L. A compendium of proteins that interact with HIF-1alpha. *Exp Cell Res* **356**, 128-135, doi:10.1016/j.yexcr.2017.03.041 (2017).

2 Kuschel, A., Simon, P. & Tug, S. Functional regulation of HIF-1alpha under normoxia--is there more than post-translational regulation? *J Cell Physiol* **227**, 514-524, doi:10.1002/jcp.22798 (2012).

3 Faubert, B. *et al.* AMPK is a negative regulator of the Warburg effect and suppresses tumor growth in vivo. *Cell Metab* **17**, 113-124, doi:10.1016/j.cmet.2012.12.001 (2013).

4 Liu, L. & Clipstone, N. A. Prostaglandin F2alpha induces the normoxic activation of the hypoxia-inducible factor-1 transcription factor in differentiating 3T3-L1 preadipocytes: Potential role in the regulation of adipogenesis. *J Cell Biochem* **105**, 89-98, doi:10.1002/jcb.21801 (2008).

5 Yun, Z., Maecker, H. L., Johnson, R. S. & Giaccia, A. J. Inhibition of PPAR gamma 2 gene expression by the HIF-1-regulated gene DEC1/Stra13: a mechanism for regulation of adipogenesis by hypoxia. *Dev Cell* **2**, 331-341 (2002).

6 Choi, S. M. *et al.* Stra13/DEC1 and DEC2 inhibit sterol regulatory element binding protein-1c in a hypoxia-inducible factor-dependent mechanism. *Nucleic Acids Res* **36**, 6372-6385, doi:10.1093/nar/gkn620 (2008).

7 Sato, F., Muragaki, Y. & Zhang, Y. DEC1 negatively regulates AMPK activity via LKB1. *Biochem Biophys Res Commun* **467**, 711-716, doi:10.1016/j.bbrc.2015.10.077 (2015).

8 Wang, M. *et al.* Uncoupling protein 2 downregulation by hypoxia through repression of peroxisome proliferator-activated receptor gamma promotes chemoresistance of non-small cell lung cancer. *Oncotarget* **8**, 8083-8094, doi:10.18632/oncotarget.14097 (2017).

9 Rieusset, J., Auwerx, J. & Vidal, H. Regulation of gene expression by activation of the peroxisome proliferator-activated receptor gamma with rosiglitazone (BRL 49653) in human adipocytes. *Biochem Biophys Res Commun* **265**, 265-271, doi:10.1006/bbrc.1999.1657 (1999).

10 Lee, J. H. *et al.* Hypoxia induces PDK4 gene expression through induction of the orphan nuclear receptor ERRgamma. *PLoS One* **7**, e46324, doi:10.1371/journal.pone.0046324 (2012).

11 Schito, L. & Semenza, G. L. Hypoxia-Inducible Factors: Master Regulators of Cancer Progression. *Trends Cancer* **2**, 758-770, doi:10.1016/j.trecan.2016.10.016 (2016).

12 Semenza, G. L. HIF-1: upstream and downstream of cancer metabolism. *Curr Opin Genet Dev* **20**, 51-56, doi:10.1016/j.gde.2009.10.009 (2010).

13 Mwaikambo, B. R., Yang, C., Chemtob, S. & Hardy, P. Hypoxia up-regulates CD36 expression and function via hypoxia-inducible factor-1- and phosphatidylinositol 3-kinase-dependent mechanisms. *J Biol Chem* **284**, 26695-26707, doi:10.1074/jbc.M109.033480 (2009).

14 Greer, S. N., Metcalf, J. L., Wang, Y. & Ohh, M. The updated biology of hypoxia-inducible factor. *EMBO J* **31**, 2448-2460, doi:10.1038/emboj.2012.125 (2012).

15 Bhattacharya, S. *et al.* Functional role of p35srj, a novel p300/CBP binding protein, during transactivation by HIF-1. *Genes Dev* **13**, 64-75 (1999).

16 Yin, Z. *et al.* The essential role of Cited2, a negative regulator for HIF-1alpha, in heart development and neurulation. *Proc Natl Acad Sci U S A* **99**, 10488-10493, doi:10.1073/pnas.162371799 (2002).

17 Ponugoti, B. *et al.* SIRT1 deacetylates and inhibits SREBP-1C activity in regulation of hepatic lipid metabolism. *J Biol Chem* **285**, 33959-33970, doi:10.1074/jbc.M110.122978 (2010).

18 Martinez-Zamora, A. *et al.* Defective Expression of the Mitochondrial-tRNA Modifying Enzyme GTPBP3 Triggers AMPK-Mediated Adaptive Responses Involving Complex I Assembly Factors, Uncoupling Protein 2, and the Mitochondrial Pyruvate Carrier. *PLoS One* **10**, e0144273, doi:10.1371/journal.pone.0144273 (2015).
19 Dogan, S. A. *et al.* Tissue-specific loss of DARS2 activates stress responses independently of respiratory chain deficiency in the heart. *Cell Metab* **19**, 458-469, doi:10.1016/j.cmet.2014.02.004 (2014).
20 Reinecke, F., Smeitink, J. A. & van der Westhuizen, F. H. OXPHOS gene expression and control in mitochondrial disorders. *Biochim Biophys Acta* **1792**, 1113-1121, doi:10.1016/j.bbadis.2009.04.003 (2009).
21 Meseguer, S. *et al.* microRNA-mediated differential expression of TRMU, GTPBP3 and MTO1 in cell models of mitochondrial-DNA diseases. *Sci Rep* **7**, 6209, doi:10.1038/s41598-017-06553-w (2017).
